# CoRE-ATAC: A deep learning model for the functional classification of regulatory elements from single cell and bulk ATAC-seq data

**DOI:** 10.1371/journal.pcbi.1009670

**Published:** 2021-12-13

**Authors:** Asa Thibodeau, Shubham Khetan, Alper Eroglu, Ryan Tewhey, Michael L. Stitzel, Duygu Ucar

**Affiliations:** 1 The Jackson Laboratory for Genomic Medicine, Farmington, Connecticut, United States of America; 2 The Jackson Laboratory, Bar Harbor, Maine, United States of America; 3 Institute for Systems Genomics, University of Connecticut Health Center, Farmington, Connecticut, United States of America; 4 Department of Genetics and Genome Sciences, University of Connecticut Health Center, Farmington, Connecticut, United States of America; University of Wisconsin, Madison, UNITED STATES

## Abstract

*Cis*-Regulatory elements (*cis*-REs) include promoters, enhancers, and insulators that regulate gene expression programs *via* binding of transcription factors. ATAC-seq technology effectively identifies active *cis*-REs in a given cell type (including from single cells) by mapping accessible chromatin at base-pair resolution. However, these maps are not immediately useful for inferring specific functions of *cis*-REs. For this purpose, we developed a deep learning framework (CoRE-ATAC) with novel data encoders that integrate DNA sequence (reference or personal genotypes) with ATAC-seq cut sites and read pileups. CoRE-ATAC was trained on 4 cell types (n = 6 samples/replicates) and accurately predicted known *cis*-RE functions from 7 cell types (n = 40 samples) that were not used in model training (mean average precision = 0.80, mean F1 score = 0.70). CoRE-ATAC enhancer predictions from 19 human islet samples coincided with genetically modulated gain/loss of enhancer activity, which was confirmed by massively parallel reporter assays (MPRAs). Finally, CoRE-ATAC effectively inferred *cis*-RE function from aggregate single nucleus ATAC-seq (snATAC) data from human blood-derived immune cells that overlapped with known functional annotations in sorted immune cells, which established the efficacy of these models to study *cis*-RE functions of rare cells without the need for cell sorting. ATAC-seq maps from primary human cells reveal individual- and cell-specific variation in *cis*-RE activity. CoRE-ATAC increases the functional resolution of these maps, a critical step for studying regulatory disruptions behind diseases.

## Author summary

Non-coding DNA sequences serve different functional roles to regulate gene expression. For these sequences to be active, they must be accessible for proteins to bind and carry out specific regulatory functions. Even so, mutations or other regulatory events may modulate their activity or regulatory function, making it critical to infer their function to understand their regulatory impact. Current sequencing technologies capture accessible sequences from low cell numbers, enabling the study of clinical samples. However, determining their functional role remains a challenge. For example, enhancers and insulators serve distinct regulatory functions, yet both fall in open chromatin regions and have similar genomic annotations (i.e., distance to transcription start site). Hence, alternative data sources and features (e.g., from DNA sequence or ATAC-seq data) must be integrated to distinguish them. Towards this goal, we developed CoRE-ATAC to infer whether open chromatin regions correspond to promoters, enhancers, or insulators. We demonstrate that CoRE-ATAC can infer regulatory functions in diverse cell types, capture activity differences modulated by genetic mutations, and can be applied to single cell ATAC-seq data to study rare cell populations. These inferences will further our understanding of how genes are regulated and how these regulatory mechanisms are disrupted with disease.

This is a *PLOS Computational Biology* Methods paper.

## Introduction

*Cis*-Regulatory Elements (*cis*-REs) are non-coding DNA sequences that can be bound by transcription factors (TFs) and can take on different functional roles (e.g., promoter, enhancer, or insulator) to regulate gene expression programs. Over 88% of disease associated single nucleotide polymorphisms (SNPs) from genome-wide association studies (GWAS) are within non-coding region of the genome [1]. In particular, these SNPs typically fall within cell-specific enhancer sequences and indirectly disrupt gene expression programs [2,3]. It is therefore critical to map *cis*-REs and their functions with increased precision to study how GWAS SNPs disrupt gene regulation in different cell types. Furthermore, genetic variation can impact the activity level of *cis*-REs [4–7]. Uncovering the functionality of such genetically-modulated *cis*-REs will help to guide *in-vivo* and *in-vitro* functional studies, by prioritizing genomic loci that are most likely to impact gene regulation for experimental validation. Furthermore, identifying *cis*-RE functions in clinical samples will help uncover individual-specific and disease-associated elements and their functional roles in pathogenesis.

ENCODE [8] and Roadmap [9] consortia successfully annotated *cis*-REs for 127 reference human cell/tissue types by profiling their epigenomes and analyzing them using a hidden markov model (HMM) based approach: ChromHMM [10]. ChromHMM integrates Chromatin Immunoprecipitation with sequencing (ChIP-seq) profiles of multiple histone modification marks and TFs to demarcate promoters, enhancers, insulators and other *cis*-REfunctions. These reference epigenomes are valuableresources to uncover and study disease-relevant and cell-type-specific *cis*-REs. However, these maps serve as references and do not capture individual- or condition-specific (e.g., activated cells) *cis*-REs. Moreover, these references do not include *cis*-REs of less frequently studied and/or rarer cells that are gaining attention with the advances in single cell profiling techniques. Although ChromHMM is very effective in inferring *cis*-RE function, it requires five or more ChIP-seq assays to be generated from the same sample, which is not always feasible due to the cost (both antibody and sequencing) and cell numbers required for ChIP-seq assays (10^4^−10^6^ cells per experiment).

**Fig 1:**
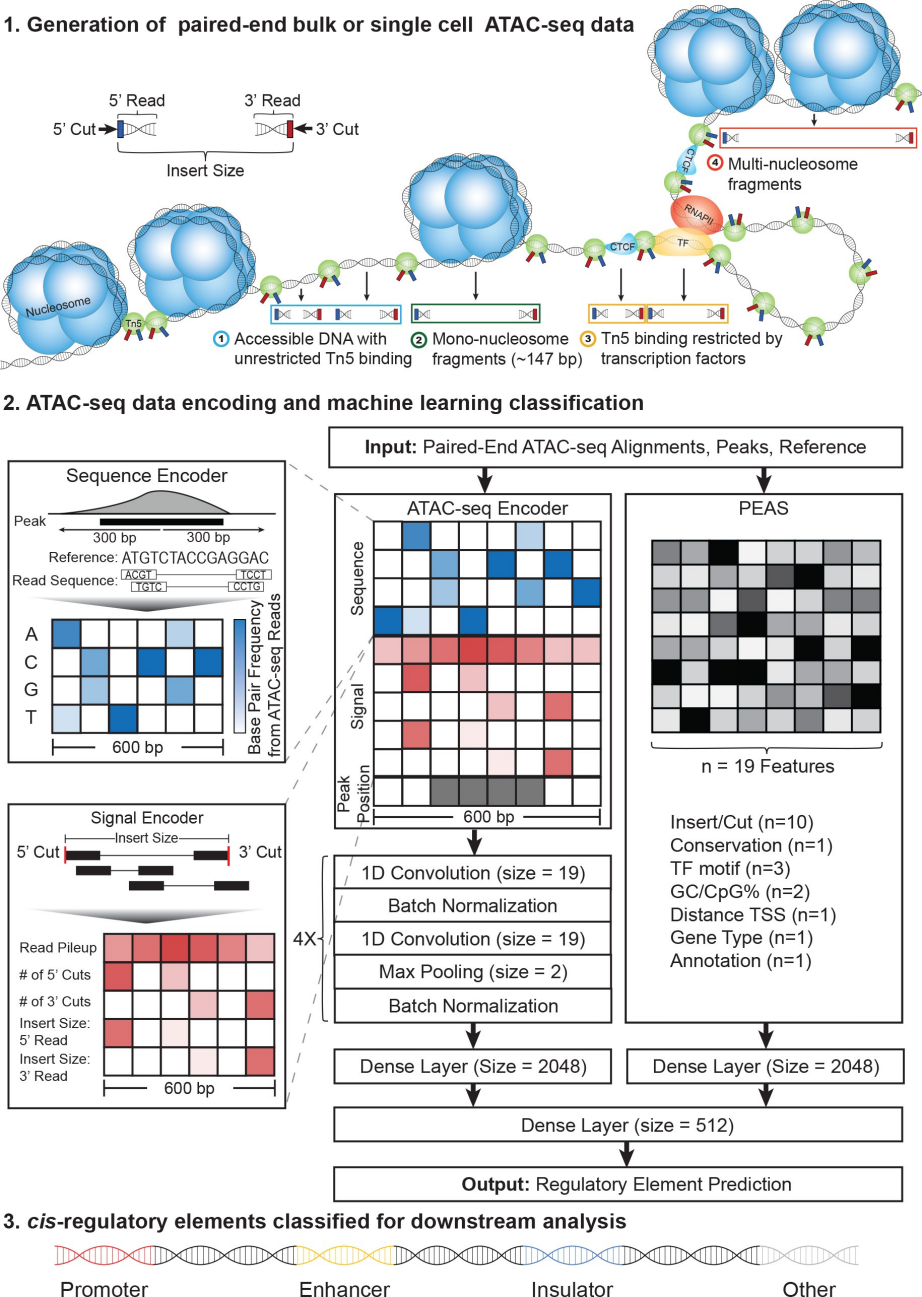
Overview of the CoRE-ATAC framework. Paired-end ATAC-seq data captures different cut and insert size distributions corresponding to the presence or absence of nucleosomes or TFs. ATAC-seq data is encoded into a 10x600 matrix and 19 data features from PEAS algorithm to predict the functionality of an open chromatin region, using both novel and manually selected features. In the final step, CoRE-ATAC classifies *cis*-REs into 4 functional classes: promoter, enhancer, insulator, and other.

An alternative strategy for genome-wide interrogation of *cis*-REs is through chromatin accessibility profiling, which identifies open chromatin regions that are accessible for binding of TFs or other regulatory proteins/RNAs. One of the most recent and most frequently used methods for identifying open chromatin regions is Assay for Transposase Accessible Chromatin using Sequencing (ATAC-seq) [11,12]. ATAC-seq utilizes Tn5 transposase to cleave accessible DNA into fragments, which are sequenced to generate a genome-wide map of open chromatin (Fig 1 Top). The low input material needed to generate ATAC-seq libraries allows it to be applied in clinical samples, making it an ideal assay for studying *cis*-REs in individual epigenomes in health and disease. Furthermore, recent developments have enabled the generation of high-quality ATAC-seq maps from single cells (i.e., single nucleus ATAC-seq (snATAC)) [13] and generate epigenomic maps from clinical samples of limited quantity. Advances in snATAC-seq technology enables researchers to study chromatin accessibility maps at unprecedented resolution by detecting rarer cell types and by studying epigenomes of cells without the need to sort them, which could affect gene expression programs. Despite these promises of snATAC-seq data, there are not many studies that show that machine learning models built from bulk ATAC-seq data can also be used for predictions on aggregated snATAC-seq data. ATAC-seq is widely used to infer *cis*-REs from diverse cell types and cell states [7,11,14–19], however, thefunctionality of these *cis*-REs (e.g., enhancers, promoters, insulators) cannot be inferred from these assays without the help of new computational methods that can fully interrogate ATAC-seq data features and integrate it with DNA sequence features.

Previously, we developed PEAS [20], a machine learning method that predicts enhancers by first extracting data features (n = 24) and then predicting enhancer activity using a neural network (NN) model. Although PEAS proved to be effective in predicting enhancers from ATAC-seq maps, it primarily utilized manually extracted features; potentially missing novel predictive features that could be hidden within the read count/cut site distributions of ATAC-seq data. Determining which features to extract for better enhancer predictions is a key challenge imposed when using this classical machine learning approach. Furthermore, PEAS was primarily developed to predict enhancer sequences, leaving the open question of whether other types of *cis*-REs can be predicted from ATAC-seq maps.

Deep learning has revolutionized the machine learning field due to its ability to learn novel data features and has been successfully applied in genomics to predict chromatin accessibility [21] and enhancers [22,23] from DNA sequence. Instead of manually extracting features from the data, deep learning can identify the most predictive features directly. To this end, we developed a deep learning framework, Classification of Regulatory Elements with ATAC-seq (CoRE-ATAC) (Fig 1 middle and bottom) which harness the power of deep learning to infer the regulatory function(s) of open chromatin regions and to overcome the limitations of our previous work, PEAS [20]. Core-ATAC integrates DNA sequence data with chromatin accessibility data (single cell or bulk) using a novel ATAC-seq data encoder that is designed to be able to integrate an individual’s genotype to personalize *cis*-RE predictions, especially for loci with genetically modulated regulatory activity (e.g., eQTLs, caQTLs). In situations where it is not feasible to generate multiple ChIP-seq assays, CoRE-ATAC can effectively infer three active functional states (promoters, enhancers, and insulators) solely from ATAC-seq data. Despite the fact thatCoRE-ATAC does not fully capture all chromatin states that can be inferred *via* ChromHMM annotations, it captures three of the most important/relevant regulatory element types responsible for transcriptional regulation and chromatin architecture.

In this study, we describe CoRE-ATAC and evaluate its ability to predict *cis*-RE function (i.e., promoters, enhancers, insulators, and others) in 11 different cell types across 46 bulk ATAC-seq samples/replicates (Table 1). We demonstrate that CoRE-ATAC is a robust method that consistently predicts *cis*-RE annotations with high average precision (mean micro average precision = 0.8, mean micro average F1 score = 0.7) irrespective of whether the cell type was used in model training. CoRE-ATAC predictions recapitulated ChromHMM enhancers from the cognate cell type as well as *cis*-REs inferred *via* different assays (CAGE [24,25] and STARR-seq [26]). We compared CoRE-ATAC *cis*-RE predictions in human islet samples to *cis*-RE activity from massively parallel reporter assays (MPRA) [27] to demonstrate that CoRE-ATAC can predict the loss/gain of *cis*-RE activity linked to genetic variation. Finally, we showed that models built from bulk ATAC-seq data are also predictive on cell clusters from snATAC-seq data, by predicting *cis*-REs from snATAC-seq data in human Peripheral Blood Mononuclear Cells [13] for 7 blood-derived immune cell type clusters. Enhancers inferred from PBMC snATAC-seq data captured the majority of super enhancers in these immune cell subsets [2,28] (i.e., enhancers that tend to be cell-type-specific) and tend toharbor SNPs for diseases of the cognate cell/tissue type. CoRE-ATAC’s ability to distinguish enhancers from insulators or other open chromatin regions is instrumental for the functional interpretation of open chromatin regions and their remodeling with age and diseases. For example, enhancers play a major role in regulating gene expression programs and tend to harbor disease-causing SNPs [2,3]. On the other hand, insulators are major players in the three-dimensional (3D) organization of the genome and help establish and maintain this 3D structure [29]. An improved functional annotation of open chromatin regions is critical for data interpretation especially while studying epigenetic remodeling in disease-relevant primary human cells and tissues. These analyses demonstrate the potential of CoRE-ATAC to annotate open chromatin regions from both bulk ATAC-seq and cell clusters from snATAC-seq samples, which will ultimately improve our understanding of how gene expression programs are regulated at the individual and cell-specific level and how this regulation is disrupted in pathologies.

**Table 1:** ATAC-seq samples used in model training and evaluation.

Cell Type	Usage	# Replicates	Data Accession Id
CD14^+^	Model Training	2	EGAS00001002605
K562	Model Training	2	GSE121993
HSMM	Model Training	1	GSE109828
GM12878	Model Training	1	GSE47753
Pancreatic Islets	Cross-cell Predictions	19	SRP117935
Naïve CD8^+^ T	Cross-cell Predictions	6	EGAS00001002605
PBMC	Cross-cell Predictions	6	EGAS00001002605
Naïve CD8^+^ T	Cross-cell Predictions	4	GSE118189
MCF7	Cross-cell Predictions	2	GSE97583
A549	Cross-cell Predictions	1	GSE117089
CD4+ T	Cross-cell Predictions	1	GSE47753
EndoC	Cross-cell Predictions	1	GSE118588
HEPG2	Additional Cross-cell Predictions	1	ENCSR888GEN
Heart (Right Atrium)	Additional Cross-cell Predictions	1	ENCSR525XSO
Heart (Left Ventricle)	Additional Cross-cell Predictions	1	ENCSR025UEI
Testis	Additional Cross-cell Predictions	1	ENCSR493GDU
Body of Pancreas	Additional Cross-cell Predictions	1	ENCSR002JUR
Stomach	Additional Cross-cell Predictions	1	ENCSR949WGV
Liver (Right lobe)	Additional Cross-cell Predictions	1	ENCSR228KEB
Thyroid	Additional Cross-cell Predictions	1	ENCSR646GBV
Transverse Colon	Additional Cross-cell Predictions	1	ENCSR654ORD
PBMC	snATAC Predictions	1	GSE129785

## Results

### CoRE-ATAC predicts functional annotations for *cis*-REs

**Fig 2:**
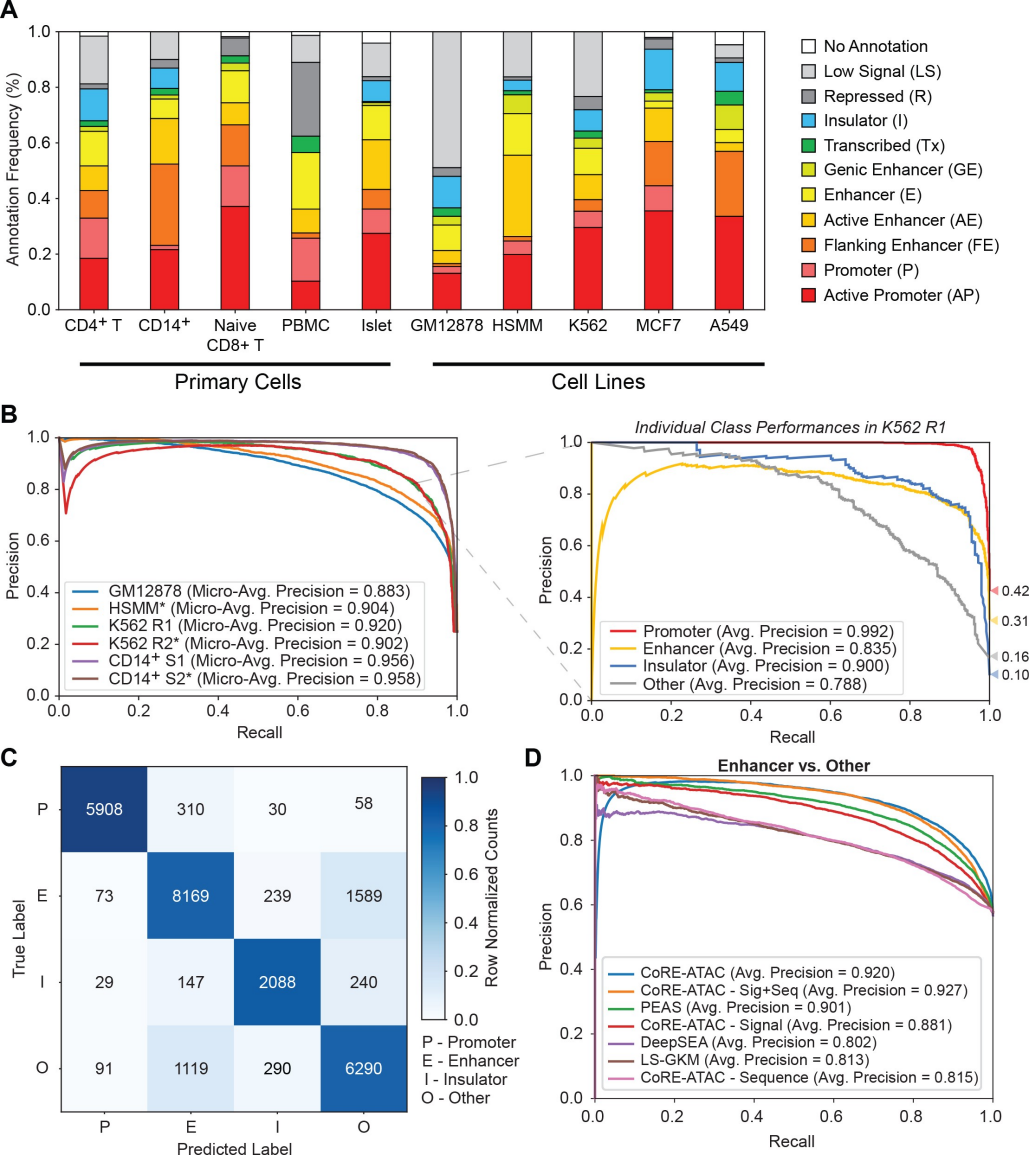
CoRE-ATAC outperforms sequence-based enhancer prediction methods. CoRE-ATAC predictions were evaluated using held out test data (chromosomes 3 and 11). (**A**) ChromHMM state distributions for different cell types used in this study. ATAC-seq open chromatin maps correspond to a multitude of *cis*-RE functional states, corresponding to Active Promoter (AP), Promoter (P), Flanking Enhancer, (FE), Active Enhancer (AE), Enhancer (E), Genic Enhancer (GE), Transcribed (Tx), Insulator (I), Repressed (R) and Low Signal (LS). (**B**) Micro-average precision values (left) were calculated, summarizing the average precision values for individual class predictions for all cell types used in model training. A breakdown of individual class average precision scores is shown for K562 (right). (**C**) Combined confusion matrix of model predictions across all cell types used in model training. Note that models are predictive for all class labels: promoters (P), enhancers (E), insulators (I), and other (O). However, mispredictions were more frequently observed between enhancer and other functional classes. (**D**) Receiver operating characteristic (ROC) curves for different enhancer prediction models: CoRE-ATAC, PEAS, DeepSEA and LS-GKM and CoRE-ATAC’s sequence, signal, and signal+sequence (No PEAS features) models. Models were evaluated for predicting enhancer versus “other” classes for chr3 and chr11 of the GM12878, HSMM, K562, and CD14+ datasets. Note that CoRE-ATAC outperforms alternative methods.

Annotation of ATAC-seq peaks using ChromHMM states revealed that between 16–52% are promoters, 18–54% are enhancers, 4–15% are insulators (for samples with insulator states in ChromHMM) and 5–55% are other functional annotations (Fig 2A). The functional diversity in these annotations establishes a need for functionally annotating ATAC-seq open chromatin maps. Furthermore ChromHMMstates are effective as baseline references to assess predictive performances of functional annotations [20] compared to alternatives (e.g., CAGE [24,25] or P300 [30,31] binding) since ChromHMM captures a wider array of functional states and detects a larger set of loci (e.g., enhancers) that can be used in model training. Previously, we showed that ChromHMM captured the majority of both P300 [30,31] and CAGE [24,25] identified enhancers, whereas P300 and CAGE enhancers identified smaller but distinct subsets of enhancers [20]. Therefore, we decided to use ChromHMM annotations as the ground truth in our models (further discussed in Discussion).

Using ChromHMM annotations in cognate cell types as class labels, CoRE-ATAC was trained on GM12878, K562, HSMM, and CD14^+^ATAC-seq samples and model performanceswere evaluated using held-out test data (i.e., regions within chromosomes 3 and 11 in the same samples) (Table 2). After identifying a concordant set of 10 functional states to use as a ground truth based on in-house and Roadmap ChromHMM states (S1 and S2 Figs), we showed that among these, four states could be effectively discriminated from ATAC-seq maps (S3 Fig). We therefore annotated ATAC-seq peaks for three major functional *cis*-RE classes (promoters, enhancers, and insulators) using ChromHMM and combined other states into a fourth class, named “other” which captures states such as repressed, transcribed, and low signal/quiescent regions, covering ATAC-seq peaks that do not correspond to the three major *cis*-REs (Materials and Methods) (S4 Fig). For the remainder of our analyses, we focus on models utilizing these four functional states: promoter, enhancer, insulator, and other.

**Table 2:** Chromosomes & number of examples for training, validation, and test data.

Category	Selected Chromosomes	Promoters	Enhancers	Insulators	Other
Training	1, 4, 5, 6, 7, 8, 9, 12, 13, 14, 15, 16, 17, 18, 19, 20, 21, 22	42554	55801	13070	41430
Validation	2, 10	6663	11361	2565	8650
Testing	3, 11	6306	10070	2504	7790

CoRE-ATAC models evaluated on 4 cell types (Table 1) had high micro-average precision (0.88–0.96) and micro-average F1 scores (0.80–0.92) across all samples with a combined accuracy of 84.20% (Fig 2B left and 2C). Individual class performances also observed high average precision for all four classes of *cis*-REs (Figs 2B right and S5), establishing that high precision/accuracy values from CoRE-ATACpredictions are not driven by predictions of a single functional class. However, we noted that most mis-predictions were between enhancer and “other” classes (Fig 2C). The “other” class is comprised of all other ChromHMM states including “transcribed”, “repressed” and “low signal” states, with the majority corresponding to the “low signal” state (S6 Fig).

To benchmark the performance of CoRE-ATAC, we compared its performance with two sequence based methods: DeepSEA [22] and LS-GKM [32], and our previous neural network based approach PEAS [20] using the same training and testing data for enhancer and “other” classes. CoRE-ATAC showed improvement over these alternative methods (Figs 2D, S7A, and S7B) (average precision = 0.92, F1 score = 0.85) for held out test data), with PEAS following second in performance (average precision = 0.90, F1 score = 0.84). Methods based solely on DNA sequence performed similarly to one another for enhancer prediction (S7A and S7B Fig) (average precision = 0.80–0.81, F1 score = 0.65–0.77) but were not as effective as PEAS and CoRE-ATAC. The increased predictive performances of CoRE-ATAC and PEAS suggest that ATAC-seq signal (e.g., read/insert pileups) contains critical information for classifying *cis*-REs that cannot be captured from DNA sequence alone. Performances for predicting promoter and insulators revealed that promoters are easily predicted by all classifiers and that CTCF insulators are difficult to detect using ATAC-seq signal alone (S7C–S7F Fig). This analysis establishes that CoRE-ATAC improves upon DNA sequence-based approaches by capturing relevant and predictive features from the ATAC-seq signal.

### CoRE-ATAC predicts *cis*-RE functionality across cell types

**Fig 3:**
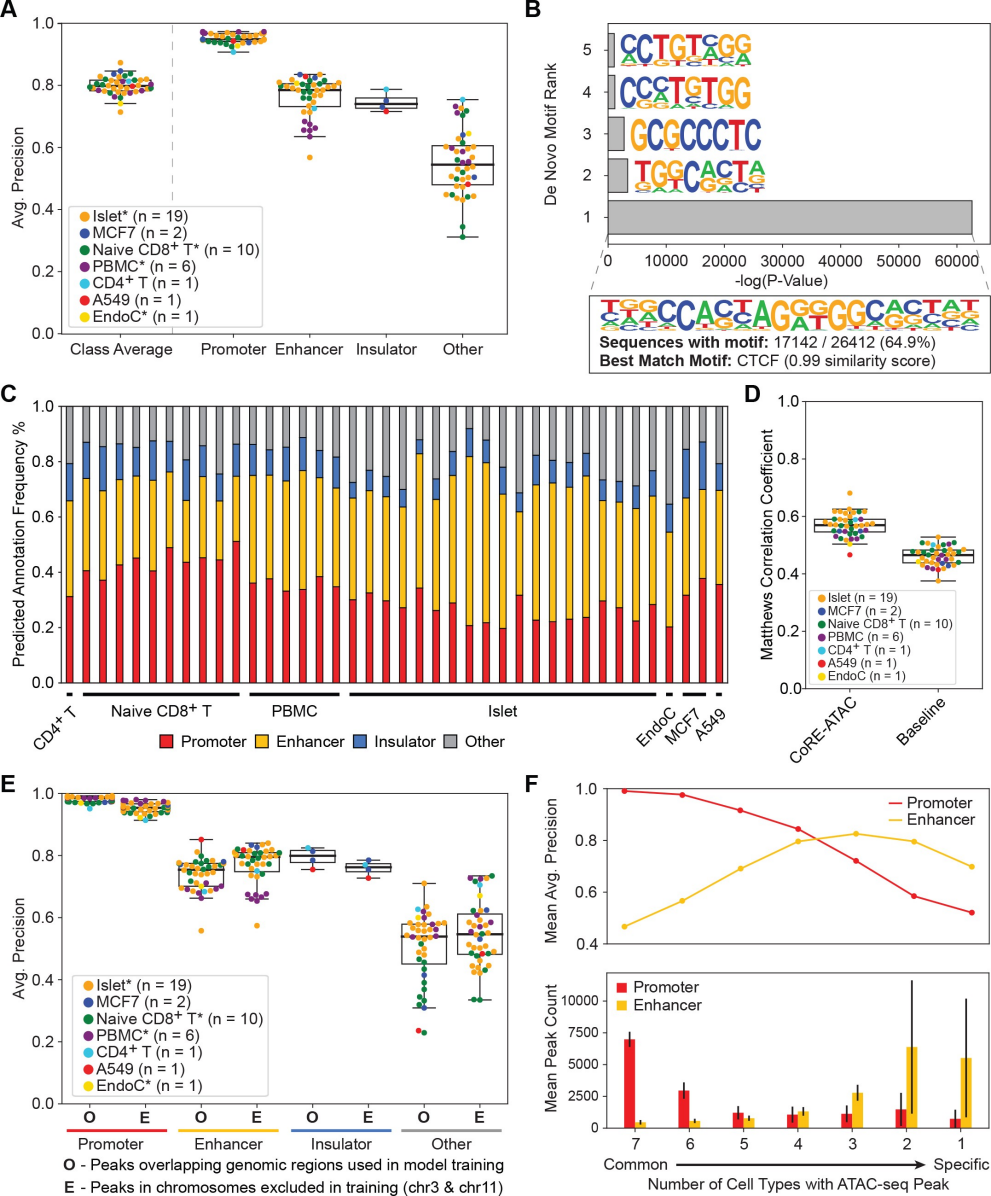
CoRE-ATAC can predict REs across cell-types. CoRE-ATAC was evaluated in 7 cell types using 40 samples that are not used in model training. (**A**) Average precision scores for predicting *cis*-REs. Micro-average precision was used to calculate class average scores. CoRE-ATAC is predictive across cell types and different functional classes with an exception of insulators in islets, which is due to CTCF ChIP-seq quality in islets. (**B**) De novo motif enrichment results for regions predicted as insulators by CoRE-ATAC but were not annotated as insulators by ChromHMM. Note that these regions are significantly enriched for the CTCF motif (0.983 similarity), suggesting that CoRE-ATAC insulator predictions are functionally relevant.(**C**) Distribution of CoRE-ATAC predictions. Prediction distributions are similar to those observed by ChromHMM state annotations. (**D**) Comparison of CoRE-ATAC to baseline/naive predictions based on thresholds for distance to TSS, MACS2 FDR, and number of CTCF motifs. CoRE-ATAC improves upon baseline performances. (**E**) CoRE-ATAC performances for i) predictions overlapping regions used in model training (O), and ii) predictions within regions that are on held-out test chromosomes (E). Note the performance similarity between these two prediction categories across all classes. (**F**) CoRE-ATAC model performances (top) and the average number of promoters and enhancers observed (bottom) by cell-type-specificity. We observed that CoRE-ATAC was more effective in predicting common promoters and cell-type-specific enhancers, for which we had more examples represented in the data. CoRE-ATAC’s ability to predict cell-type-specific enhancers demonstrates its usefulness for interrogating individual and cell-type-specific enhancers.

We evaluated CoRE-ATAC’s ability to predict cis-RE function in 40 bulk ATAC-seq samples from different cell types including four primary cells (i.e., pancreatic islets, naïve CD8+ T, PBMC, CD4+ T) and three cell lines (MCF7, A549, EndoC beta cell line). These samples were not used in model training (held-out, Table 1). Note that among these, MCF7 (breast cancer) and A549 (adenocarcinoma) are cancer cell lines; hence they have different cellular characteristics than the primary cells used in training. CoRE-ATAC models were highly predictive for cell types that were not utilized in model training, showing~0.80 micro-average precision and 0.80–0.92 micro-average F1 scores across all samples (Fig 3A). The highest precision values were detected for promoters (~0.95), followed by enhancers (~0.76) (Fig 3A). Insulator annotations were only available in 4 of the 7 cell types tested (MCF7, A549, CD4^+^ T and islet), however, insulator states in islets were excluded from these analyses due to the poor quality of CTCF ChIP-seq data in islets (S8 Fig), which affected the performance assessment for islet samples. Among the remaining 3 cell types, known insulators (based on ChromHMM states) were predicted with high average precision (~0.78) (Figs 3A and S9 for islets). As expected, the majority of insulator predictions resided within CTCF ChIP-seq peaks (S10 Fig). To further show the functional relevance of CoRE-ATAC insulators that were either not annotated by ChromHMM (i.e., cell types that are missing insulator states) or misclassified when compared to ChromHMM insulators, we conducted de novo motif enrichment analysis using HOMER [33]. CTCF motif was the most enriched sequence for these loci with a very significant p-value (P-value < 1e-27180) (Fig 3B), suggesting that CoRE-ATAC predictions capture CTCF insulator sequences. It is possible that since CoRE-ATAC relies on CTCF binding motifs to detect insulators, it might mistake non-insulator CTCF sites as insulators.

As expected, functional annotation of ATAC-seq peaks from these 40 samples *via* CoRE-ATAC (Fig 3C) were similar to state distributions observed with ChromHMM (Fig 2A). In addition, we studied whether functional predictions with CoRE-ATAC can outperform a naïve annotation approach using distance to transcription start site (TSS), MACS2 FDR, and number of CTCF motifs (Materials and Methods). CoRE-ATAC annotations outperformed these naïve annotation approaches (Figs 3D and S11), observing ~0.58 Matthews correlation coefficient score for CoRE-ATAC compared to ~0.47 on the average across all samples. Matthews correlation coefficient was used to account for the fact that threshold-based approaches produce binary probabilities (i.e. 1.0 or 0.0) that can result in misleading average precision calculations.

A potential pitfall in cross-cell-type model evaluations is the use of the same genomic regions (e.g., same chromosome) in both model training and testing [34]. When the same region (not the same genomic data) is used in training and testing, a model might perform well simply because it “remembers” the specific DNA sequences used during training. To study whether our model suffers from this pitfall, we utilized the two chromosomes that were excluded from model training (chromosomes 3 and 11) and compared cross cell type predictions for these two chromosomes with regions that overlapped loci used in model training and observed comparable predictive performances (Fig 3E). These analyses suggest that CoRE-ATAC has learned a function from DNA sequence and chromatin accessibility signals that is transferable across genomic regions for predicting *cis*-RE functionality rather than learning to memorize specific DNA sequences.

We further analyzed cross cell type performances by comparing enhancers versus other predictions of CoRE-ATAC with alternative models while incorporating cell types from 9 different tissues and cell types from ENCODE [8,35,36]: Body of Pancreas, Liver (Right Lobe), HepG2, Heart (Left Ventricle), Heart (Right Atrium), Transverse Colon, Stomach, Testis, and Thyroid. We observed that CoRE-ATAC outperformed alternative sequence-based methods(Mann Whitney P-Values < 0.0096 for ROC AUC values) for enhancer prediction while promoters and CTCF insulators were predicted with high performances for the majority of classifiers (S12 Fig). Based on these performances, CoRE-ATAC is the best performing model for predicting enhancer activity in new cell types among the models tested.

Finally, we evaluated CoRE-ATAC’s cross-cell-type performance by stratifying ATAC-seq peaks by their cell-specificityacross 7 cell types and compared prediction performances. CoRE-ATAC predicted common promoters with higher average precision than cell-type-specific promoters. Interestingly, CoRE-ATAC predicted cell-type-specific enhancers more effectively compared to common enhancers (mean average precision 0.70 versus 0.48), emphasizing the utilityof this method to study disease-relevant enhancers that are typically cell-type-specific [2,3] (Fig 3F top). The prediction bias observed for cell-specific promoters is likely due to the number of elements used in model training given that the majority of promoters are common among cell types (Fig 3F bottom), whereas the majority of enhancers are cell-type-specific.

### Core-ATAC models are robust to training with a low number of cell types

We explored whether increasing the number of cell types could improve the performance of CoRE-ATAC. For this, we trained three different models for comparisons. The first model with 7 different cell types: GM12878, HSMM, Body of Pancreas, Stomach, Thyroid, Testis, and Transverse Colon. The second model was trained using the same cell types used previously, while including Heart (Left Ventricle) and Heart (Right Atrium). Finally, the third model was trained just as the second, but including Liver and HepG2 samples. We tested these models on held out cell types and chromosomes and determined that increasing the number of cell types had little impact for CoRE-ATAC’s predictive power (S13 Fig). The original cell types used by CoRE-ATAC are likely sufficient for training the models to learn the signatures of *cis*-REs present in ATAC-seq data.

### Core-ATAC can predictenhancers that are captured *via* different assays

**Fig 4:**
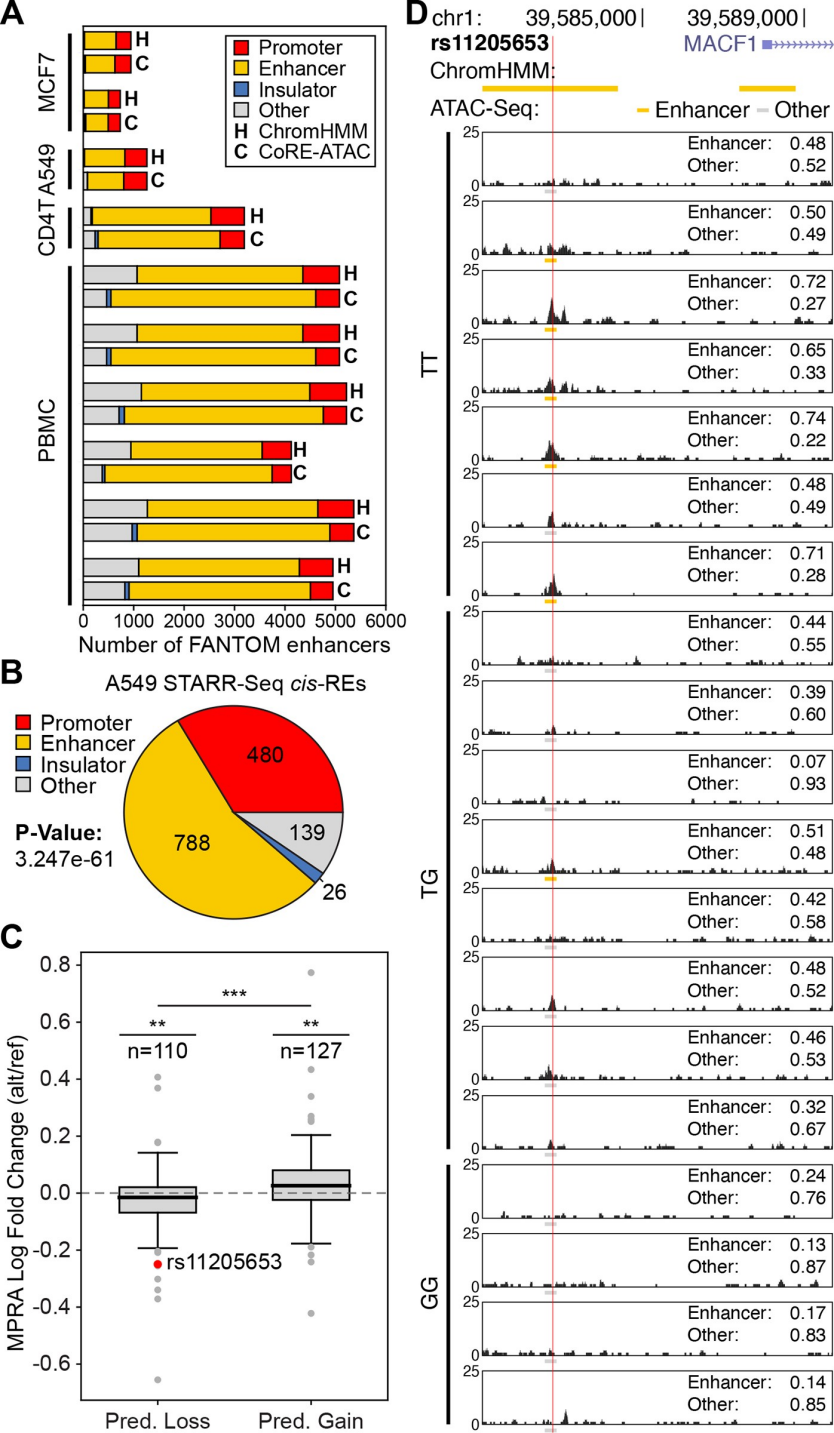
CoRE-ATAC predictions overlap with experimentally detected enhancers. (**A**) Overlap of FANTOM enhancer annotationswith CoRE-ATAC (C) and ChromHMM (H) predictions in MCF7, A549, CD4^+^ T and PBMC samples. CoRE-ATAC predicted the majority of FANTOM enhancers as enhancers or promoters, recapitulating these experimentally identified enhancers. CoRE-ATAC annotations were similar toChromHMM annotations. (**B**) CoRE-ATAC predictions for active regulatory regions identified by STARR-seq in A549 cell line. The majority of active enhancers identified by STARR-seq were predicted as promoter or enhancer by CoRE-ATAC. (**C**) MIN6 MPRA log fold change values for genomic regions predicted as losing or gaining *cis*-RE function based on CoRE-ATAC probabilities for reference and alternative alleles. Significance for predicted loss and predicted gain categories was calculated using student’s t-test for MPRA log fold change values being less than or greater than 0 respectively. Significance comparing the predicted loss and predicted gain of MPRA fold change distributions was calculated using Mann-Whitney U test. We observed concordant direction of effect both for CoRE-ATAC predictions and MPRA activity levels when alternative and reference alleles are compared. (**D**) Genome browsers of 19 islet samples highlighting a loss of enhancer activity for rs11205653 (also highlighted in (**C**)) for the alternative allele (G). Values for enhancer and other represent the probability assigned to those classes of *cis*-REs by CoRE-ATAC. We observe that for 5 out of 7 individuals with the reference allele (TT) CoRE-ATAC predicted enhancer activity, reflecting ChromHMM reference annotations, while for the individuals with GT or GG alleles, we observed an enhancer activity loss for all but one individual based on CoRE-ATAC predictions.

Several methods have been established for experimentally identifying enhancer sequences. The FANTOM5 project [37,38] generated a databaseof enhancers for over a thousand tissues and cell lines using Cap Analysis of Gene Expression (CAGE) [24,25], which identifies enhancers using the observation that balanced bidirectional capped transcripts correspond to active enhancers. More recently, massively parallel reporter assays (MPRA) [27] have enabled experimentally testing thousands of sequences in parallel for regulatory activity (i.e., promoters and enhancers) [39]. In particular, self-transcribing active regulatory region sequencing (STARR-seq) [26] identifies enhancers by cloning sequences downstream of a promoter that transcribes the enhancer sequence depending on its activity. Previous studies showed that ChromHMM captures these experimentally identified enhancers with high overlap/enrichment [20,40]. To study whether CoRE-ATAC also detects enhancers identified by alternative methods, we first compared ChromHMM annotations and CoRE-ATAC predictions in MCF7, A549, CD4^+^ T, and PBMC cell types to CAGE [24,25] enhancers identified by the FANTOM5 project [37,38]. We observed that the majority of FANTOM enhancers overlapped with promoters and enhancers in both ChromHMM and CoRE-ATAC (Figs 4A and S14A for test set chromosomes). CoRE-ATAC enhancer predictions showed significant overlap with FANTOM enhancers (Fisher’s exact test p-values for all cell types <4.52e-59 for all chromosomes, <5.29e-10 for test set chromosomes). The similarity between ChromHMM and CoRE-ATACfurther establishes that functional annotations *via* CoRE-ATAC using ATAC-seq data is a cost-effective alternative to annotations *via* ChromHMM that use multiple ChIP-seq assays.

Next, to understand whether CoRE-ATAC could detect enhancers identified *via* MPRAs, we first compared enhancer predictions in A549 cells to enhancers identified from STARR-seq [26,41]. The majority of sequences that showed regulatory activity in this assay were predicted as enhancers (overlap significance calculated using Fisher’s exact test p-value <3.247e-61 for all chromosomes, p-value <6.367e-09 for test chromosomes) by CoRE-ATAC (Figs 4B and S14B). We noted that a significant portion of STARR-seq enhancers (~33%) were predicted as promoters in our models and the majority of these regions were close to TSS (S14C and S14D Fig). In contrast, CoRE-ATAC enhancers overlapping STARR-seq active regions were more distal from the TSS (S14E and S14F Fig). CoRE-ATAC predictions resembled ChromHMM annotations for promoters and insulators (S14G and S14H Fig), 480 and 474 promoters, 26and 35 insulators for CoRE-ATAC and ChromHMM respectively (all chromosomes). However, ChromHMM has more information available allowing it to correctly annotate more enhancers (868 enhancers) than CoRE-ATAC (788 enhancers), which only uses ATAC-seq data.

Regulatory activities of certain open chromatin regions are genetically modulated, which can be detected *via* chromatin accessibility quantitative trait loci (caQTL) analyses. Previously, we identified caQTLs from human islet samples (n = 19) [7] for which we generated an MPRA library to test and compare the regulatory activity of reference and alternative alleles forcaQTLs and other variants (n = 4293 SNPs) [42]. These data gave us the opportunity to test whether CoRE-ATAC predictions can detect genetically driven differences in the regulatory activity. Using CoRE-ATAC prediction probabilities from 19 islets (stratified based on genotypes), we identified 237 loci for which a gain or loss of enhancer activity was predicted based on individuals’ genotypes and CoRE-ATAC prediction probabilities using one-tailed point-biserial correlation p-values (Materials and Methods). Among these, 110 loci lost activity in the alternative allele, whereas 127 gained activity. For these sequences, we compared the regulatory activity from MPRA assays to the predicted gain/loss of activity from CoRE-ATAC and confirmed that the direction of effect coincides with the two analyses (Figs 4C and S15 for test chromosomes only). More specifically, loci with gain of function for the alternative allele based on CoRE-ATAC predictions had higher MPRA activity for thealternativeallele in comparison to the reference allele (i.e., fold change>0). A similar concordance was observed for loci associated with loss of function for the alternative allele. For example, islet samples with the alternative allele for SNP rs11205653 (an islet caQTL) had lower enhancer probabilities compared to the samples with the reference allele, in agreement with the activity levels from the MPRA library for this locus (Fig 4D). Although CoRE-ATAC enhancer predictions were higher for individuals with TT genotype compared to individuals with GG and GT genotypes, we noted that individual-level heterogeneity in *cis*-RE activity levels within the same genotype. This heterogeneity likely stems from non-genetic factors including disease status (diabetic versus healthy) or other clinical information (i.e., medication use, sex, race). Together, these results and findings establish CoRE-ATAC’s ability to predict individual level variability in *cis*-RE activity including heterogeneity stemming from genetic variation.

### Predicting disease-relevant enhancers from single nuclei ATAC-seq data

**Fig 5:**
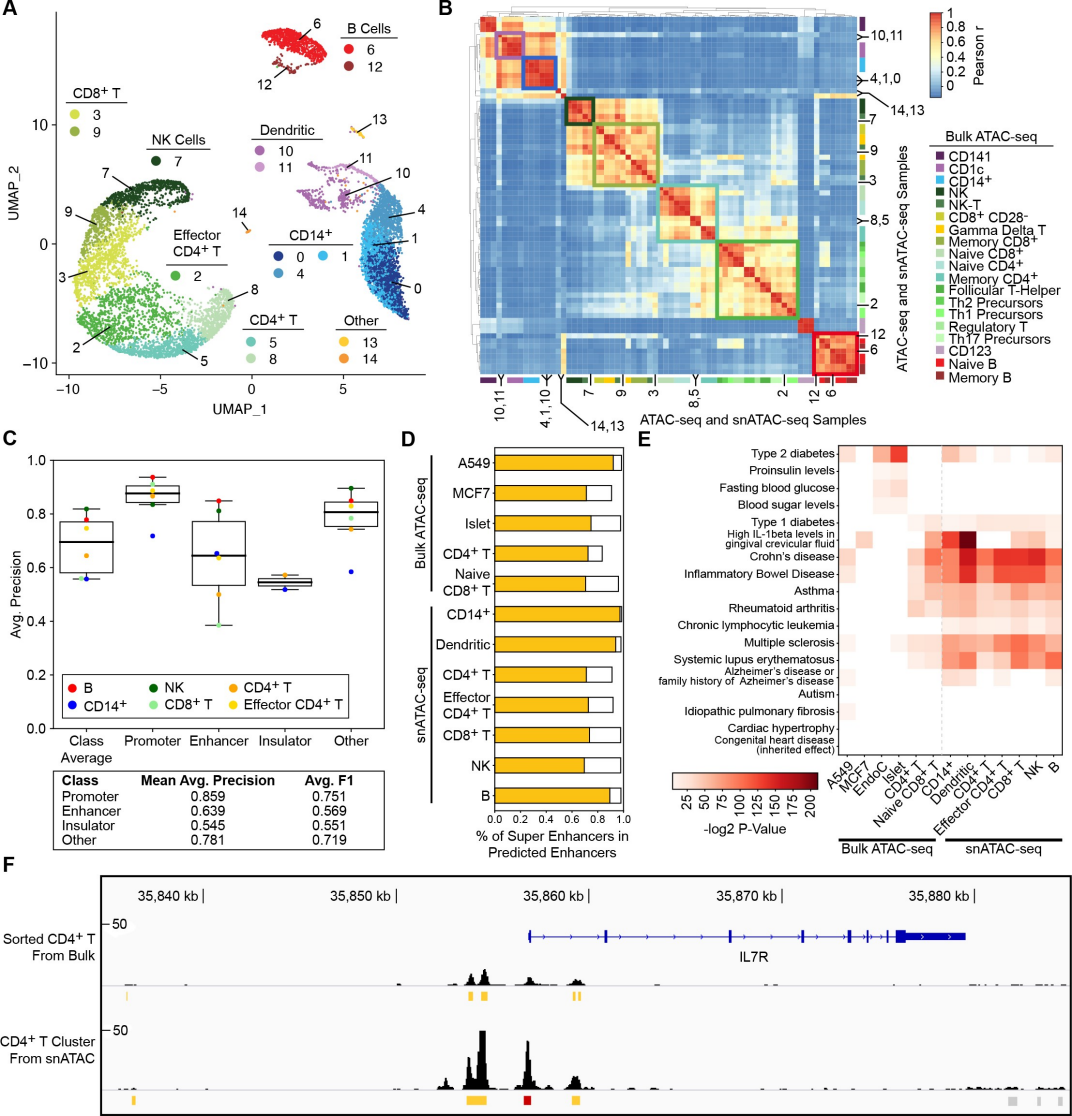
Predicting functionality of REs from clusters of PBMC snATAC-seq data. (**A**) Single cell clusters annotated for 7 immune cell types. Two-pass clustering identified a total of 15 cell clusters which we annotated using hierarchical clustering with sorted bulk ATAC-seq data (shown in (**B**)) to identify 7 different immune cells corresponding to these clusters. (**B**) Hierarchical clustering of snATACclusters with bulk ATAC-seq data. Numbers and highlighted regions within the heatmap correspond to cell clusters and annotations in (**A**). 7 immune cell types were observed with both snATAC and bulk ATAC-seq samples. (**C**) (Top) Average precision values for predicting *cis*-RE function in snATAC for 6 annotated clusters with available ChromHMM states. Model performances suggest that CoRE-ATAC is an effective tool for interrogating *cis*-RE activity from snATAC data. (Bottom) Mean average precision and average F1 score values for promoters, enhancers, insulators and other. (**D**) Percent of super enhancers detected among CoRE-ATAC enhancers, demonstratingCoRE-ATAC’s ability to identify cell-type-specific enhancers that are most relevant to disease. (**E**) GREGOR SNP enrichment analysis highlighting selected diseases whose SNPs were significantly enriched within the enhancer elements predicted by CoRE-ATAC. Enhancers from PBMCsnATAC-seq were significantly enriched for SNPs associated with immune diseases. (**F**) Genome browser view of *IL7R* for bulk ATAC and snATAC samples for CD4^+^T cells. ATAC-seq read profiles and CoRE-ATAC predictions between snATAC and bulk ATAC were found to be similar to one another, demonstrating CoRE-ATAC as a robust method for *cis*-RE predictions. Red represents promoter predictions, yellow represent enhancer predictions, and gray represent “other” predictions from CoRE-ATAC.

Single nucleus ATAC-seq (snATAC-seq)reveals chromatin accessibility at single nucleus resolution and enables 1) interrogation of chromatin accessibility at the single cell level; 2) identifying epigenomic maps of rare cell types *via* unsupervised clustering methods [13]; 3) enabling the study of cell types within tissues without the need to sort cells. In light of this emerging technology, we applied CoRE-ATAC models (trained on bulk data) to predict cis-REs in snATAC-seq data. To test this, we predicted *cis*-RE functions from human PBMC snATAC data [13] by first clustering cells into 15 groups based on the similarity of their accessibility profiles (Fig 5A). Comparisons with sorted immune cell bulk ATAC-seq data [14,17] revealed 7 distinct cell types corresponding to B, natural killer (NK), CD8^+^ T, CD4^+^ T, effector CD4^+^ T, CD14^+^, and dendritic cells (DCs) (Fig 5B). CoRE-ATAC models trained on bulk ATAC-seq data, predicted *cis*-REfunction in snATAC aggregate clusters (mean micro-average precision = 0.68, mean micro-average F1 score = 0.41) (Figs 5C and S16), showing the flexibility and robustness of the method. Insulator predictions from snATAC-seq data were significantly enriched for CTCF/BORIS motifs (S17 Fig), confirming the biological relevance of these predictions.

Similar to enhancer predictions in bulk ATAC-seq, CoRE-ATAC enhancer predictions in snATAC clusters also included cell-specific enhancers for immune cell subsets within PBMCs (S18 Fig). Cell-type specific peaks identified by CoRE-ATAC in these immune cells significantly overlapped with cell-specific enhancers inferred from ChromHMM annotations of relevant immune cell types (Materials and Methods) (S19 Fig). Super enhancers tend to be cell type-specific and often overlap disease-relevant SNPs [2]. We therefore further studied CoRE-ATAC’s ability to identify super enhancers in these 7 immune cell types obtained from SEdb [43]. On average, ~81% of super enhancers were captured by CoRE-ATAC enhancer predictions within their respective cell types for single cell predictions (Fig 5D), which was comparable to the detection rate from bulk ATAC-seq data which captured ~76% of super enhancers. The ability to identify cell-specific enhancers from snATAC-seq data is instrumental for studying rare cell populations and clinical samples.

We further assessed CoRE-ATAC’s ability to identify disease-relevant enhancers by conducting GWAS SNP enrichment analyses using GREGOR [44].For this analysis we comparedCoRE-ATAC enhancer prediction in the 7 cell types inferred from snATAC-seq data with enhancers predicted from bulk ATAC-seq data (A549, MCF7, islets, EndoC, CD4^+^ T and naïve CD8^+^ T). Immune cell enhancers predicted from snATAC-seq data were enriched in diseases related to the immune system (Fig 5E). For example, B cell enhancers were the most significantly enriched for variants linked to Systemic Lupus, a disease characterized by dysfunctions in B cells [45]. Islet enhancers were most significantly enriched for Type 2 Diabetes and Fasting Blood Glucose as expected, while T cell were most significantly enriched in immune diseases (e.g., Type 1 Diabetes). Overall, enhancer predictions were enriched in diseases that were the most relevant to their respective cell types, confirming that CoRE-ATAC can infer cell-specific enhancers. Immune cell enhancers predicted from snATAC-seq data were enriched in diseases related to the immune system (Fig 5E) and were the most enriched among the other classes of predictions (S20 Fig) as expected.

Chromatin accessibility profiles of snATAC-seq clusters resembled that of bulk data from sorted cells, enabling CoRE-ATAC to effectively predict *cis*-RE function from snATAC-seq data, e.g., the locus around the *IL7R* (an important gene for T cell aging [14]) in CD4^+^ T cells both in bulk and single cell maps (Fig 5F). Despite similarities between sorted cell and single cell cluster epigenomes, the efficacy of CoRE-ATAC on snATAC-seq data was not given due to the differences in how cells areprocessed (i.e., FACS sorted cells versus single cell clusters) as well as differences between bulk and snATAC-seq libraries (depth, peak sizes, read distributions etc.). Our analyses have demonstrated that CoRE-ATAC models built from bulk data can effectively predict *cis*-RE function in snATAC-seq data, which is essential for the analyses of future snATAC-seq maps.

## Discussion

Recent advances in ATAC-seq profiling and single cell genomics revolutionized the epigenomics field by enabling the generation of chromatin accessibility maps from small starting material, even at single cell resolution. Due to these advances, epigenomic maps of human cells/tissues from many individuals are being generated at an unprecedented rate, including our own studies, to study how epigenomic landscapes change with age, diseases, and upon in-vivo and in-vitro activation [7,11,14–19]. These epigenomic maps are instrumental for inferring *cis*-REs from clinically relevant samples. CoRE-ATAC harnesses the power of deep learning to integrate chromatin accessibility maps with DNA sequence and effectively predicts the functionality of *cis*-REs (i.e., promoters, enhancers, insulators) across diverse cell types and individuals. We extensively evaluated CoRE-ATAC’s efficacy to predict *cis*-RE function across multiple cell types including those that are not used in model training. We established that CoRE-ATAC is an effective method for classifying the functional state of *cis*-REs, in new cell types (i.e., cell types that are missing reference annotations) and in snATAC-seq data.

One of the unique features of CoRE-ATAC is its ability to integrate an individual’s genotype with chromatin accessibility maps by inferring the genotype from ATAC-seq read alignments. CoRE-ATAC accomplishes this with a sequence encoder that uses the frequency of ATAC-seq reads observing a specific base-pair at a genomic position instead of solely using a one hot encoding of the reference genome, separating our method from existing ones that use the commonly used one hot-encoding approach. If genotype data (e.g., SNP arrays) are available for an individual, one can also leverage this information in CoRE-ATAC predictions by providing a reference specific to the individual. Genotype-aware methods can be effective in predicting the annotation and activityof *cis*-REs whose activity is modulated by genetic variation (e.g., caQTLs [4–7]). In alignment with this, we demonstrated that CoRE-ATAC predictions from individual islet epigenomes aligned with enhancer loss/gain between alleles inferred from MPRA assays. CoRE-ATAC’s ability to improve enhancer predictions at the individual level is studied at islet caQTLs, i.e., regions for which variation at the chromatin accessibility levels are associated with the genetic variation. Hence, the good performance of CORE-ATAC for these loci stem from both ATAC-seq signal as well as the underlying sequence since these two modalities are correlated with each other.

CoRE-ATAC also established a foundation for inferring functional annotations from snATAC-seq clusters, which can be useful to study rare cell populations that can be identified from these assays. For this analysis, we used CoRE-ATAC models trained on bulk data due to current limitations on the availability of snATAC-seq data that coincide with existing ChromHMM/*cis*-RE annotations. Although models from bulk data were effective in predicting functionality of *cis*-REs from single cell data, models built from snATAC-seq data might further improve the predictive performance. In the future, as more snATAC-seq data becomes available for more samples and more diverse cell types with established ChromHMM and CTCF insulator states, a CoRE-ATAC model can be trained solely on snATAC-seq data, potentially resulting in better classification performances for the purposes of annotating rare cell populations. Our predictions from snATAC-seq data were conducted at the cluster level. Given the sparsity of chromatin accessibility information at the individual single cell level (open in both alleles, open in one allele, closed), predicting functionality at the single cell level will remain a challenge.

Although CoRE-ATAC can effectively predict several functional classes of cis-REs, it has several limitations. First, CoRE-ATAC is limited to discriminating promoters, enhancers, and insulators, while categorizing the remaining open chromatin regions as ‘Other’. The ‘Other’ category includes multiple ChromHMM states (i.e., “repressed”, “transcribed”, “low signal”). Data features that we extract from chromatin accessibility maps and DNA sequence alone may not be sufficient to further annotate these loci. Second, despite the fact that CoRE-ATAC can capture enhancer activity in an individualized manner, since CoRE-ATAC uses both ATAC-seq maps and DNA sequence for predictions, it may sometimes fail to learn the importance of DNA sequences that are especially correlated with ATAC-seq signal patterns (e.g., caQTLs). Hence, it can fail to detect SNP effects on chromatin accessibility levels; more appropriate methods should be used to detect these effects [5]. Finally, CoRE-ATAC was trained using chromatin states defined by ChromHMM [10], which is another computational inference method. ChromHMM utilizes more and different types of data (i.e., multiple ChIP-seq assays) and does not use ATAC-seq maps to infer different chromatin states. The unsupervised approach of ChromHMM identifies clusters of genomic regions that have similar combinations of transcription factor/histone modification marks which can then be annotated using domain knowledge (e.g., states with H3K4me1, H3K27ac are likely enhancers). Although ChromHMM states are not directly and experimentally established cis-RE functions, they have multiple advantages over alternatives for class labeling in our machine learning models: 1) ChromHMM profiles are genomewide providing many examples for model training, 2) ChromHMM states are available for many different cell types, enabling training and testing models across many cell/tissue types, and 3) ChromHMM states are well studied and functionally validated [46], making these annotations high-quality references, despite being computational inferences. In terms of capturing enhancer function, we previously showed that when overlapping ChromHMM enhancers with FANTOM [37,38] and P300 [30,31] enhancers, ChromHMM captured the majority of both of these sets of enhancers [20]. Another study compared ChromHMM with STARR-seq enhancers, and showed that STARR-seq enhancers [26] were significantly enriched for ChromHMM enhancer states [40]. We therefore decided that ChromHMM annotations were the most comprehensive functional annotations for training our deep learning models.

CoRE-ATAC is widely applicable for inferring *cis*-RE function from ATAC-seq and further improves upon naïve methods as well as existing machine-learning models (Figs 2D and 3B) through its ability to leverage ATAC-seq signal information. Although model training is a computationally expensive process, requiring GPUs to train in a timely manner, using these models for predictions is much faster, requiring a little over 2 minutes (excluding the time to load the data into memory) to functionally annotate *cis*-REs for 75000 loci using a 2.3 GHz 8-Core Intel Core i9 processor (S21 Fig). Our in-depth performance analyses suggest thatCoRE-ATAC can be widely used, even with limited computational resources, to improve the functional annotations of ATAC-seq *cis*-RE maps. To promote the widespread use of our predictive model we have made the CoRE-ATAC code and pre-trained model freely available on GitHub (https://github.com/UcarLab/CoRE-ATAC).

## Materials and methods

### Machine learning architecture

CoRE-ATAC utilizes both data encoded in the deep learning framework and features extracted using our previous method PEAS [20]. These two feature sets are provided as two separate inputs into amachine learning model implemented using Keras [47] (version 2.2.4) and Tensorflow [48] libraries. The deep learning component uses four convolutional layer blocks that are trained with a batch size of 32. Each block consists of i) a 1D convolutional neural network (CNN) layer (window size = 19, stride = 1), ii) a batch normalization layer, iii) a second 1D CNN layer (window size = 19, stride = 1) iv) a max pooling layer (pool size = 2, stride = none) and v) a second batch normalization layer, in this order (Fig 1). The first two blocks of convolutional layer units utilize 256 filters while the final two blocks utilize 512 filters. Both the deep learning component and the PEAS component of the model are trained separately using their own dense neural network layer with n = 2048 nodes. For each component, default initialization was used to train the models without any prior knowledge. The outputs of these dense layers are then saved, concatenated, and provided as input into a combined dense layer with n = 512 nodes before classifying *cis*-REs in the final output dense layer using the Adam [49] optimization methodwith the default learning rate (0.001). CNN layer window sizes, number of filters, and number of dense layer nodes were selected based on the best parameters observed during model tuning.

### ATAC-seq data encoders

We implemented a novel ATAC-seq data encoder that takes as input: paired-end reads, peaks, and the reference genome (or a personalized genome reflecting an individual’s genetic variation) to generate one 10x600 matrix per peak. Rows represent DNA sequence and ATAC-seq signal data, while columns represent the 300 base-pair positions downstream and upstream from the peak center (Fig 1). Details of this matrix are explained below:

***Rows 1–4*:** The 0–1 normalized frequency of observing 1) adenine (A), 2) cytosine (C), 3) guanine (G), or 4) thymine (T) from ATAC-seq read pileups, which is calculated when there is enough read coverage (n > = 10). For low coverage loci; the frequency reduces to a one-hot encoding, representing the corresponding nucleotide in the reference genome.
***Row 5*:** The number of insert pileups within the 600 base-pair window, where each position is z-standardized with respect to pileups across all peaks for that position.
***Rows 6 and 7*:** The number of 5’ (row 6) and 3’ (row 7) cuts at a given position (z-standardized). Savitzky-Golay smoothing filter [50] is applied with a window size of 15 to account for an increase in the number of cuts across multiple positions in close proximity due to sequencing depth.
***Rows 8 and 9*:** The median fragment length for 5’ and 3’ cut site, applying the same standardization and smoothing used for rows 6/7.
***Row 10*:** The original peak region, setting values to 1 if a base-pair position is within the peak and 0 otherwise. This allows for distinguishing between peaks that are in close proximity to one another and providing an indicator of the most relevant positions.

In all analyses, peaks used for data encoding were called with MACS2 [51] using parameters “-f BAMPE—nomodel”. Duplicates were kept only in the snATAC-seq data analyses using the “—keep-dup all” option.

### ATAC-seq data processing

Paired end ATAC-seq reads were processed using ATAC-seq pipeline available at https://github.com/UcarLab/ATAC-seq. First, read adapters were trimmed using Trimmomatic [52] (version 0.33). Next, reads were aligned to the hg19 reference genome using BWA MEM [53] (version 0.7.10-r789 for GM12878, CD14^+^, and islets, version 0.7.12-r1039 for all other samples). Finally, duplicates were marked using the Picard Toolkit [54] (version 2.8.1 for EndoC, version 1.95 for all other samples) and removed. snATAC-seq data was processed using CellRangerATAC (version 1.2) using default parameters.

### Ground truth selection& model training

CoRE-ATAC was trained on ATAC-seq data from 4 different cell types: GM12878 [11] (GSE47753), K562 [19] (GSE121993), HSMM [15] (GSE109828, “54–1” at time 0), and CD14^+^ monocytes [14] (EGAS00001002605) (Table 1). Class labels were assigned by co-analyzing 15-state ChromHMM [10] models generated in these cell types with corresponding 18-state Roadmap [9] ChromHMM annotations. 15 state ChromHMM models were trained for each cell type independently using H3K4me1, H3K4me3, H3K27ac, H3K27me3, H3K36me3, H3K9me3 and CTCF ChIP-seq data from ENCODE [8] (https://genome.ucsc.edu/ENCODE/index.html). Hierarchical clustering of emission probabilities revealed 10 distinct clusters (S1 Fig) corresponding to active promoters, promoters, flanking enhancers, active enhancers, enhancers, genic enhancers, transcribed, insulator, repressed, and low signal states. Preliminary machine learning models were trained using the 10 clusters identified and peaks were selected based on their concordance with Roadmap [9] 18 state models (S2 Fig) in order to select the best examples for model training. Performance evaluations on the 10-state models revealed that only 4 major classes of *cis*-REs could be effectively predicted from such models across all cell types: promoter, enhancer, insulator and other classes (S3 Fig). Based on these results, we focused on training models with these 4 major classes and selected peaks using a wider range of concordance with Roadmap states (S4 Fig).

While training CoRE-ATAC, we envisioned two use case scenarios for annotations 1) use of a single ATAC-seq sample; 2) use of a set of samples (i.e., multiple islet ATAC-seq samples) co-analyzed together. In the latter case, consensus peaks would be obtained from multiple samples and need to be annotated. In our experiments using consensus peaks, we noted that some “Other” regions are not called as peaks in all the samples, although they were among the consensus peaks. This could reflect individual-level heterogeneity and/or noise in either the data or peak calling step. To train a model that is robust to such instances, we incorporated non-peak regions for half of the samples (3 out of 6): HSMM, CD14+ Sample 2, and K562 replicate 2. Non-peak regions annotated as “Other” were selected among the consensus peaks that are not called as a peak in that cell type. Normalization steps are performed on each sample individually when encoding the data. Importantly, to not overwhelm the model with non-peak examples (since some of it could be noise), we restricted these examples to be at most 25% of all “Other” regions in that sample. In total, only 2,551 out of 57,870 (~4.4%) “Other” cases were such non-peaks. This step was implemented to help classify instances of non-peaks from a sample in case they are introduced to the algorithm, (i.e., *via* consensus peak data analyses.)

Deep learning and PEAS components of CoRE-ATAC were initially trained separately to handle different rates of overfitting observed between the two models (overfitting evaluated using training and validation loss). Parameter tuning was performed in this stage of training to adjust i) the number of filters, ii) convolutional layer window size, iii) number of convolutional blocks, and iv) number of nodes within dense layers (where applicable) using held out validation data (i.e., ATAC-seq peaks in chromosomes 2 and 10) (Table 2). Individually, each component achieved 82.56% and 79.25% accuracy on held out validation data for deep learning and PEAS components respectively using the best observed parameters for minimizing the validation loss. We then combined these models into a single unified model by concatenating deep learning and PEAS components with a concatenation layer, for a final round of model training.

### Model tuning

Hyperparameter selection was performed on i) the 1D convolution kernel size {9, 11, 19, 21}, ii) the number of convolutional layer block {3,4,5}, iii) intermediate dense layer size {1024, 2048, 4096} and iv) and the number of convolutional layer filters {32, 128, 256} where for each block the first two convolutional layers were set to the initial filter size and then doubled for the remaining two convolutional layers. Due to the long training time on these models, not every parameter combination (108 total) was tested. Instead, different parameters were tested one by one, and the best performing value for the parameter was kept. It is therefore possible that a more rigorous search for optimal hyperparameters could further improve CoRE-ATAC’s performance.

### Model evaluation

To test the performance of CoRE-ATAC on datasets and cell types that were not used in model training, we initially predicted *cis*-REs in ATAC-seq data obtained for 7 different cell types: MCF7 [16] (GSE97583) (n = 2), Naïve CD8^+^ T [14,17] (GSE118189 & EGAS00001002605) (n = 10), Peripheral Blood Mononuclear Cells (PBMCs) [14,17] (EGAS00001002605) (n = 6), CD4^+^ T [11] (GSE47753) (n = 1), A549 [55] (GSE117089) (n = 1), pancreatic islets [7] (SRP117935) (n = 19) and EndoCbeta cell line [18] (GSE118588) (n = 1) (Table 1). We later measured the performance of CoRE-ATAC on an additional 9 cell types from ENCODE [8,35,36]: HEPG2 [56] (ENCSR888GEN), Heart (Right Atrium) [57] (ENCSR525XSO), Heart (Left Ventricle) [58] (ENCSR025UEI), Testis [59] (ENCSR493GDU), Body of Pancreas [60] (ENCSR002JUR), Stomach [61] (ENCSR949WGV), Liver (Right lobe) [62] (ENCSR228KEB), Thyroid [63] (ENCSR646GBV), and Transverse Colon [64] (ENCSR654ORD). Model performances were evaluated using ChromHMM states in each cell type.

### ChromHMM annotations for model evaluation

For the majority of datasets, 15 state ChromHMM models were trained using H3K4me1, H3K4me3, H3K27ac, H3K27me3, H3K36me3, H3K9me3, and CTCF (when available) ChIP-seq data obtained from public repositories (Table 3). For the remainder, ChromHMM states were used directly from their respective studies for EndoC [18] and the additional cell types identified for snATAC-seq clusters (closest references) which were obtained from Roadmap [9] (https://egg2.wustl.edu/roadmap/web_portal/, 18 state models): B Cells (E032), NK Cells (E046), CD8^+^ T (E048, Note: Memory CD8^+^ T was selected as the closest reference), and Effector CD4^+^ T (E043, Note: CD4+, CD25-,Th was selected as the closest reference). Recalled ChromHMM states were converted to the 4 major class labels used by CoRE-ATAC based on emission probability correlations with the 10 cluster previously identified (S1 Fig). The final ChromHMM 4 state labels are annotated in S8 Fig EndoC states (using state labels from the study [18])were converted based on the following: promoters were mapped from states labeled as 1_Active_TSS, 2_Weak_TSS, and 14_Bivalent_poised_TSS, enhancers were mapped from states labeled as 8_Genic_enhancer, 9_Active_enhancer_1, 10_Active_enhancer_2, and 11_Weak_enhancer, and other were mapped from states labeled as 5_Strong_transcription, 6_Weak_transcription, 16_Repressed_polycomb, 17_Weak_repressed_polycomb, and 18_Quiescent_low_signal. For 18 state models used directly from Roadmap [9]: promoters were mapped from states labeled as 1_TssA, 2_TssFlnk, enhancers were mapped from states labeled as 7_EnhG1, 8_EnhG2, 9_EnhA1, 10_EnhA2, and 11_EnhWk, and other were mapped from states labeled as 5_Tx, 6_TxWk, 12_ZNF/Rpts, 13_Het, 16_ReprPC, 17_ReprPCW, 18_Quies. EndoC and Roadmap ChromHMM data did not include CTCF insulators; hence some of the insulators in these cell types are likely mislabeled as other states. This is a limitation of using CoRE-ATAC on these datasets.

**Table 3:** ChromHMM References.

Cell Type	Database	Data Accession Id/URL
CD14^+^	ENCODE (UCSC)	http://hgdownload.soe.ucsc.edu/goldenPath/hg19/encodeDCC/wgEncodeBroadHistone/
K562	ENCODE (UCSC)	http://hgdownload.soe.ucsc.edu/goldenPath/hg19/encodeDCC/wgEncodeBroadHistone/
HSMM	ENCODE (UCSC)	http://hgdownload.soe.ucsc.edu/goldenPath/hg19/encodeDCC/wgEncodeBroadHistone/
GM12878	ENCODE (UCSC)	http://hgdownload.soe.ucsc.edu/goldenPath/hg19/encodeDCC/wgEncodeBroadHistone/
Naïve CD8^+^ T	ENCODE Portal	ENCSR465PPP
MCF7	ENCODE Portal	ENCSR247DVY
A549	ENCODE Portal	ENCSR797CXN
CD4+ T	GEO	GSE17312 (Histone Marks) [65], GSE12889 (CTCF) [66]
PBMC	GEO	GSE16368 (from TC015) [65]
Pancreatic Islets	GEO	GSE51312 [3], GSE23784 [67]
EndoC	GEO	GSE118588 [18]
Heart (Right Atrium)	ENCODE Portal	ENCSR525XSO
Heart (Left Ventricle)	ENCODE Portal	ENCSR025UEI
Testis	ENCODE Portal	ENCSR493GDU
Body of Pancreas	ENCODE Portal	ENCSR002JUR
Stomach	ENCODE Portal	ENCSR949WGV
Liver (Right lobe)	ENCODE Portal	ENCSR228KEB
Thyroid	ENCODE Portal	ENCSR646GBV
Transverse Colon	ENCODE Portal	ENCSR654ORD
B Cells	Roadmap	E032
NK Cells	Roadmap	E046
Memory CD8^+^ T	Roadmap	E048
CD4^+^, CD25^-^,Th	Roadmap	E043
ENCODE (UCSC)	https://genome.ucsc.edu/ENCODE/downloads.html	
GEO	https://www.ncbi.nlm.nih.gov/geo/	
ENCODE Portal	https://www.encodeproject.org/	
Roadmap	https://egg2.wustl.edu/roadmap/web_portal/	

ChromHMM annotates the genome by assigning states for every 200bp interval. Consecutive states are merged by ChromHMM, and the size of these state assignments varies based on the number of times the same state is called in succession (i.e., multiples of 200). ChromHMM states were then relabeled to Promoter, Enhancer, Insulator, or Other, based on their emission probabilities and our annotations (e.g., S8 Fig). We overlapped these states with the ATAC-seq peaks that are of different lengths. To eliminate ambiguous annotations in our models, we only used ATAC-seq peaks that were unanimously (90% of its entirety) assigned to a single functional state among the 4 states: Promoter, Enhancer, Insulator, or Other. Filtering by 90% overlap with a single class label removed an average of 4002 peaks per sample, ranging from 0.3–16.2% of the total number of peaks per sample.

### Comparison with alternative methods/assays

We compared CoRE-ATAC to two sequence-based methods (DeepSEA [22] and LS-GKM [32]) and our previous neural network (NN) based method (PEAS [20]). For this comparison we focused on enhancer versus “other” predictions, the most difficult discrimination task in our models (Fig 2C), and compared all annotated regions from our ground truth test set (i.e., regions within chromosomes 3 and 11 for GM12878, K562, HSMM, and CD14^+^ samples) (Tables 1 and 2). The same training and testing set were used for CoRE-ATAC, PEAS, and LS-GKM. For DeepSEA, we used the web annotation tool (https://hb.flatironinstitute.org/deepsea/). DeepSEA makes multiple predictions of activity for a wide array histone marks and transcription factors (TFs) across multiple cell types. We therefore selected enhancer probabilities by taking the maximum probability score for H3K4me1 and H3K27ac across all cell types predicted by DeepSEA. For promoters, probability scores were selected using H3K4me3 and pol2. For insulators, probability scores were selected using CTCF. The area under the receiver operating characteristic curve (ROC AUC) and average precision metrics were used to compare the four methods (Figs 2D and S7.).

Naïve method comparisons focused on the 40 samples not used in model training to fairly assess CoRE-ATAC’s performance with respect to these methods. Multiple thresholds were applied for each Naïve method to identify the best threshold to set for predicting promoters, enhancers, and insulators. Promoters were tested using 1kb, 2kb, and 5kb distances to the nearest TSS, (distances calculated using HOMER [33]). Enhancers were tested using MACS2 [51] FDR qval of 0.01, 0.001, and 0.0001. Insulators were tested using the number of CTCF motifs greater than 0, 2, and 4. Promoters, enhancers, and insulators were selected using these thresholds, assigning probabilities 1.0 when the threshold requirement is met, and 0.0 otherwise. Finally, combined naïve approach performance was calculated selecting the best threshold (i.e., 2kb, 0.001, and 0 for promoter TSS, enhancer MACS2 qval, and insulator number of CTCF motifs respectively). Due to the nature of selecting enhancers using MACS2 qval, priority was given to promoters and then insulators. For the remaining regions that were not annotated as either promoter or insulator, enhancers and “other” were classified using MACS2 qval. Performances were evaluated using Matthews correlation coefficient.

Alternative enhancer definitions were explored to understand how well CoRE-ATAC can predict active regulatory elements (i.e., promoter and enhancers) from FANTOM [37,38] and STARR-seq [26]. FANTOM enhancers identified using Cap Analysis of Gene Expression (CAGE) technology [24,25] were obtained for MCF7, A549, CD4^+^ T cells, and PBMCs. We obtained STARR-seq active regulatory sites for A549 [41] from the Gene Expression Omnibus (GEO) [68,69] (GSE114063). These regions were then compared with CoRE-ATAC predictions, counting the number of predictions for each class within FANTOM5 enhancers and STARR-seq sites. Fisher’s exact test was used to calculate the significance of whether a peak was identified as an enhancer by CoRE-ATAC, FANTOM/STARR-seq, or both (i.e, using a 2x2 contingency table between CoRE-ATAC and FANTOM/STARR-seq enhancer annotations). All remaining non-enhancer ATAC-seq peaks were used as the background in these calculations.

Massively Parallel Reporter Assay (MPRA) data were generated in MIN6 pancreatic beta cell line to study the regulatory activity of variants associated with Type-2-Diabetes [42]. Briefly, 4293 variants within islet ATAC-seq peaks were profiled for regulatory element activity using MPRA. Taking the union of islet peaks called across all 19 samples, we predicted *cis*-RE functions for each islet sample to obtain class probabilities for each region and islet sample. We then putatively identified *cis*-REs showing loss or gain of *cis*-RE activity using one-tailed point-biserial correlation p-values, identifying loci with probabilities that were significantly lower or higher between reference and alternative genotypes. The maximum absolute value correlation was obtained for each peak with genotype information by calculating the correlations for all comparisons among ref/ref, ref/alt, and alt/alt genotypes, using the sum of promoter and enhancer probabilities. Peaks with significant point-biserial correlation coefficients (p-value < 0.01) were separated into two groups corresponding to the loss or gain of *cis*-RE activity (negative and positive correlations respectively) based on CoRE-ATAC predictions for different alleles. Finally, MPRA activity differences between alternative and reference alleles (log fold change in MPRA analyses) were compared with the activity differences inferred from CoRE-ATAC *cis*-RE class probabilities. Student t-test, and Mann Whitney U test were used to calculate the significanceofMPRA log foldchange values observed for predicted loss and gain of *cis*-RE activity both individuallyas well as comparatively.

### snATAC seq data analyses and clustering

Single nuclei ATAC-seq (snATAC) PBMC data [13] was obtained from GEO [68,69] (GSE129785). Sequence reads were processed using Cell Ranger ATAC and cells were clustered using our own implementation of a recently described snATAC clustering method [13], which uses two passes of clustering to identify cell type clusters. In the first pass, genomic regions were binned into 2500bp windows, counting the number of paired-end reads within each bin. The top 50,000 bins with the greatest number of reads were selected for clustering using Seurat [70,71], requiring a minimum of 185 cells per cluster. Peaks were called for each cluster independently using MACS2 (“-f BAMPE–nomodel,—keep-dup all”) and were used to perform a second pass of cell clustering as before, using peaks instead of bins, identifying a total of 15 clusters.

Cell type annotations for these clusters were obtained by comparing snATAC profiles with ATAC-seq profiles of sorted immune cell types*via* flow cytometry. To identify marker peaks for each cell type, we selected 2–3 representative and high quality ATAC-seq samples per cell type [14,17] for 19 different immune cell types based on ATAC-seq library quality (FRIP score and read depth) and similarity between biological replicates (Spearman r). Marker peaks were identified from the signature profile generated by CIBERSORT [72], using its data pre-processing step. Hierarchical clustering was performed for read pileups within marker peaks for snATAC-seq clusters and sorted bulk data, which enabled annotating 15 clusters into 7 cell types. Cells that belong to the same cell type were pooled together and the functionality of *cis*-REs were predicted using CoRE-ATAC for 7 cell types by allowing duplicate reads. Predictions were evaluated using either ChromHMM annotations from Roadmap or in-house ChromHMM states (CD4^+^ T and CD14^+^ monocytes).

Comparison of cell-specific enhancers were calculated using Fisher’s exact test. First, cell-specific ATAC-seq peaks were identified from union peaks by merging peaks called from all snATAC-seq clusters. Cell-specific peaks were identified by counting the number of overlapping peaks sets for each union peak. CoRE-ATAC and ChromHMM enhancers were identified from these cell-specific peaks, and Fisher’s exact test was applied to calculate the significance of CoRE-ATAC cell-specific enhancers overlapping with ChromHMM cell-specific enhancers. All remaining non-enhancer peaks were used as the background for these calculations.

### SNP enrichments

GWAS SNP enrichments were performed with GREGOR [44] software using index SNPs as well as linked SNPs (linkage disequilibrium threshold of R^2^ 0.7 for the European (EUR) population). NHGRI-EBI GWAS Catalog SNPs [73] (Obtained January 8^th^ 2020) for 3981 traits/diseases were used in enrichment analyses for different enhancer sets inferred by CoRE-ATAC.

### Super enhancer analysis

Super enhancer annotations were obtained for A549, MCF7, Islets, CD14^+^, CD4^+^ T, CD8^+^ T, monocyte-derived dendritic cells, NK, and B cells from SEdb [43]. We then calculated the percent of super enhancers found among CoRE-ATAC enhancer predictions to measure how well CoRE-ATAC predictions recapitulate these cell-type-specific enhancers.

## Supporting information

S1 Fig ChromHMM emission probability clusters reveal 10 consistent chromatin states. We clustered the ChromHMM emission probabilities using Pearson’s correlation coefficient to identify 10 chromatin states that were consistently present among all 10 cell types for which we recalled ChromHMM states. These 10 functional states include, insulators, repressed, low signal, enhancer, active enhancers, active promoter, flanking enhancer, promoter, genic enhancer, and transcribed regions. Numbers preceding the cell types are the emission states corresponding to S8 Fig. Note that genic enhancers are shown by two groups which correspond to active genic enhancers and genic enhancers which we combined due to the low number of cell types included within these clusters independently. Also note that for insulators, we selected islet state 7 as this was the state with the strongest CTCF signal (S8 Fig), maintaining one insulator state per cell type.
(TIF)

S2 Fig CoRE-ATAC 10 State model ground truth selection. To select a ground truth for predicting the 10 functional states we identified, we corroborated our ChromHMM state calls with Roadmap 18 state models. (Top) Highlighted concordant recalled ChromHMM states with corresponding Roadmaps states to select active promoters (red), promoters (pink), flanking enhancers (orange), active enhancers (orange yellow), enhancers (yellow), genic enhancers (greenish yellow), transcribed (dark green), insulator (blue), repressed (dark gray), and low signal (light gray) functional states. (Bottom) The number of ground truth examples for each cell type used in model training and functional state examples selected. Note: Roadmap 18-state models do not include insulator states and we therefore chose insulators from our ChromHMM models that included CTCF ChIP-seq data. These predicted insulators mostly overlap with the Quiescent state in the 18-state models, suggesting that this ChromHMM state (Quies) is a miscellaneous functional state in the absence of CTCF ChIP-seq.
(TIF)

S3 Fig CoRE-ATAC 10 State performances. (**A**) Confusion matrices of combined CoRE-ATAC performances for 10 state models. (**B**) Confusion matrices of CoRE-ATAC performance for 10 state models in GM12878. CoRE-ATAC 10-state models. Only 4 of the 10 chromatin states were predicted by CoRE-ATAC. Smaller subsets of these four functional states (i.e., promoters, enhancers, insulators and other) were predicted as the state with the highest number of examples.
(TIF)

S4 Fig CoRE-ATAC 4 State model ground truth selection. (Top) Highlighted concordant recalled ChromHMM states with corresponding Roadmaps states to select promoters, enhancers, insulators and other. (Bottom) The number of ground truth examples for each cell type used in model training and functional state examples selected. Flanking enhancers were excluded as these regions are ambiguous and could be annotated as promoters. Merging states and relaxing concordance with Roadmap allowed for selecting more examples for model training. Note: Roadmap 18-state models do not include insulator states and we therefore chose insulators from our ChromHMM models that included CTCF ChIP-seq data. These predicted insulators mostly overlap with the Quiescent state in the 18-state models, suggesting that this ChromHMM state (Quies) is a miscellaneous functional state in the absence of CTCF ChIP-seq.
(TIF)

S5 Fig CoRE-ATAC predicts all functional annotations with high precision. Precision recall curves of held out test data for each sample used in model training. Individual class performances reveal that CoRE-ATAC predicts all classes with high average precision.
(TIF)

S6 Fig Majority of “Other” peaks are low signal. (Left) The number of peaks identified as Transcribed, Repressed, or Low Signal by ChromHMM states (blue), overlayed with the number of these peaks predicted as “Other” by CoRE-ATAC. (Right) The fraction of Transcribed, Repressed or Low Signal states annotated as “Other” by CoRE-ATAC. The majority of these states (>50% for each state, >69% overall) are predicted as “Other”.
(TIF)

S7 Fig CoRE-ATAC component comparisons with sequence-based enhancer predictions. Receiver operating characteristic (ROC) curves (**A**) and Precision Recall curves (**B**) for different enhancer prediction models: CoRE-ATAC components, PEAS, DeepSEA and LS-GKM. Models were evaluated for predicting enhancer versus “other” classes for chr3 and chr11 of the GM12878, HSMM, K562, and CD14+ datasets. Sequence based approaches had similar performances, including CoRE-ATAC’s sequence-based component. The ATAC-seq signal based (CoRE-ATAC-Signal) component of CoRE-ATAC alone outperforms all sequence-based approaches, however combining both sequence and signal greatly enhances predictive performances. PEAS captures more information than signal alone, however, is still underperforming compared to CoRE-ATAC-Sig+Seq model. Finally, the CoRE-ATAC model, which includes PEAS features, showed a slight improvement over the CoRE-ATAC-Sig+Seq model, likely taking advantage of the known features such as number of known motifs and conservation scores used in PEAS. (**c**) ROC AUC and (**d**) average precision scores for predicting promoters in chr3 and chr11. All models perform with high ROC AUC and Average Precision for predicting promoters due to distinct signatures for both sequence and ATAC-eq signal. (**e**) ROC AUC and (**f**) average precision scores for predicting insulators in chr3 and chr11. DNA sequence is the approaches are the best performing models for predicting CTCF insulators, while ATAC-seq signal alone provides the weakest predictive power for annotating these *cis*-REs.
(TIF)

S8 Fig ChromHMM emission probabilities for all cell types. Recalled ChromHMM states for 10 of the 11 cell types used in this study. Each chromHMM run included enhancer marker H3k4me1, promoter marker H3k4me3, repressor marker H3k27me3, active *cis*-RE marker H3k27ac, transcribed marker H3k36me3, heterochromatin marker H3k9me3, and CTCF insulator marker CTCF (when available). Emission probabilities revealed consistent histone modification mark combinations present throughout these diverse cell types. Color annotations next to each heatmap of ChromHMM states represent the inferred ChromHMM state and the final 4 state relabeling.
(TIF)

S9 Fig CoRE-ATAC Islet insulator predictions. (**A**) Cross cell type model performances when including islet insulators. Using poor quality CTCF ChIP-seq data (as evident from S8 Fig) as a ground truth resulted in reduced model performances for all islet samples. (**B**) De novo motif enrichment for CoRE-ATAC insulator predictions in islets. Predicted insulators are highly enriched for CTCF with 76.1% of regions harboring a CTCF motif. Incorporating both DNA sequence and ATAC-seq signal enables CoRE-ATAC to detect a majority of insulators using the CTCF motif while detecting other insulators *via* other features learned in model training.
(TIF)

S10 Fig CTCF enrichment of CoRE-ATAC insulator predictions. Overlap of CTCF ChIP-seq peaks with CoRE-ATAC insulator and non-insulator predictions. Majority of CoRE-ATAC insulator predictions overlapped with CTCF ChIP-seq peaks. Note: Overlaps between insulator and non-insulators are the result of CTCF peaks bridging the gap between these genomic regions when merging peaks for the union.
(TIF)

S11 Fig CoRE-ATAC outperforms threshold-based naïve/baseline annotations. CoRE-ATAC outperforms each threshold-based method for detecting promoters, enhancers, insulators, and combined annotations. Matthews Correlation Coefficient was used to measure performances as it accounts for true positives, true negatives, false positives, and false negatives simultaneously within its function and is an ideal measurement for performances when probabilities are static as in the case with all threshold-based approaches (i.e., probability = 1.0 if satisfying the threshold, 0.0 otherwise). CoRE-ATAC consistently had the best performance compared to each baseline. For detecting promoters, the best threshold was identified as ATAC-seq peaks within 2kb of the promoter, for enhancers, an FDR qval score is less than 0.0001, and for insulators, whether or not the region contained a single (n = 1) CTCF motif was the best performing threshold. Finally, combining all three of these thresholds confirmed that CoRE-ATAC improves our ability to predict *cis*-RE function, outperforming commonly used methods for detecting promoters, enhancers, and insulators.
(TIF)

S12 Fig Performance of alternative methods on cell types that are not used in training. (**A**) ROC AUC, (**B**) average precision, (**C**) F1, and (**D**) recall scores for predicting enhancers in chr3 and chr11 across different cell types not used in model training. CoRE-ATAC generalizes well for predicting enhancers in new cell types. CoRE-ATAC outperforms alternative sequence-based models (Mann Whitney P-Values over ROC AUC < 0.0096). Note: The model for DeepSEA was trained using MCF7, A549, Stomach, Heart Left Ventricle, Heart Right Atrium, Pancreas, Liver, HEPG2, CD8 Naïve T Cells, and CD4+ T cells.(**E**-**H**) Performance scores for predicting promoters in chr3 and chr11 across different cell types not used in model training. All models predict promoters with high accuracy, irrespective of cell type. (**I**-**L**) Performance scores for predicting insulators in chr3 and chr11 across different cell types not used in model training. DeepSEA outperforms other methods for detecting CTCF insulators. CTCF has a well characterized DNA binding sequence that is very predictive.
(TIF)

S13 Fig CoRE-ATAC performance with different number of cell types used in training. Three CoRE-ATAC models were trained using: 1) 7 different cell types: GM12878, HSMM, Pancreas, Stomach, Thyroid, Testis, and Transverse Colon, 2) 9 different cell types: 7 cell type model with 2 Heart samples (Left Ventricle and Right Atrium Auricular Region) and 3) 11 different cell types: 9 cell type model with Liver and HepG2 samples. CoRE-ATAC performances did not significantly improve with increased number of cell types used for training for promoter, enhancer, or other prediction. Insulator prediction showed significant differences between models (Mann Whitney P-Values < 1.91e-06, Average Precision), however, the effect of these differences suggests that the model decreased in performance as more data was used for model training.
(TIF)

S14 Fig CoRE-ATAC predicted enhancers significantly overlap FANTOM and STARR-seq enhancers. (**A**) Distribution of CoRE-ATAC predictions for FANTOM enhancers in test chromosomes (chr3 and chr11). (**B**) Distribution of CoRE-ATAC predictions for test chromosomes. Pairs of bars represent comparisons between ChromHMM (H) and CoRE-ATAC (C), where each pair represents a sample/replicate for the respective cell type. (**C**,**D**) Histogram of distances to the nearest TSS for STARR-seq enhancers predicted as promoters by CoRE-ATAC for all chromosomes (**C**) and test chromosomes (**D**). Majority of predicted promoters are within 1kb of a TSS. (**e**,**f**) Histogram of distances to the nearest TSS for STARR-seq enhancers predicted as enhancers by CoRE-ATAC for all chromosomes (**E**) and test chromosomes (**F**). Majority of predicted enhancers are distal (> = 20kb) from the nearest TSS. STARR-seq enhancers annotated as promoters result from the close proximity these enhancers are to a TSS. (**G**,**H**) ChromHMM annotation (relabeled to 10 classes) distribution for STARR-seq enhancers in A549 for all chromosomes (**G**) and test chromosomes (**H**). Majority of STARR-seq enhancers are annotated as promoter or enhancer by ChromHMM.
(TIF)

S15 Fig CoRE-ATAC predicts direction of effect for loci not observed in model training. MIN6 MPRA log fold change values for genomic regions predicted as losing or gaining cis-RE function based on CoRE-ATAC probabilities for reference and alternative alleles in test chromosomes (chr3 and chr11). Significance for predicted loss and predicted gain categories was calculated using student’s t-test for MPRA log fold change values being less than or greater than 0 respectively. Significance comparing the predicted loss and predicted gain of MPRA fold change distributions was calculated using Mann-Whitney U test. Concordant direction of effect (P-Value < 0.022) was observed for both for CoRE-ATAC predictions and MPRA activity levels for chromosomes not used in model training.
(TIF)

S16 Fig CoRE-ATAC predicts *cis*-REs in snATAC-seq cell type clusters. Average precision, ROC AUC, F1 score, and recall for predicting cis-RE function in snATAC for 6 annotated clusters with available ChromHMM states. Model performances suggest that CoRE-ATAC is an effective tool for interrogating cis-RE activity from snATAC data.
(TIF)

S17 Fig Predicted insulators in snATAC data are enriched for CTCF. Top de novo motif enrichments for all seven cell type clusters for snATAC data. Insulator predictions for all cell types are significantly enriched for motifs with high similarity with CTCF.
(TIF)

S18 Fig CoRE-ATAC predictions identify cell specific enhancers in snATAC-seq data. Number of enhancers identified for the top 25 cell types and combinations by the number of enhancers predicted by CoRE-ATAC.
(TIF)

S19 Fig Comparison of CoRE-ATAC and ChromHMM cell-specific enhancers. Overlap of CoRE-ATAC andChromHMM cell-specific enhancers. CoRE-ATAC predictions significantly overlap ChromHMM enhancers for all cell types (P-Values < 3.85e-190 Fisher exact test). Overlaps were less significant for T cell comparisons, partially due to the differences in sorted cells used in ChromHMM annotations and total cells used in CoRE-ATAC predictions from snATAC-seq data.
(TIF)

S20 Fig GREGOR SNP Enrichments for CoRE-ATAC predictions. Selected disease SNP enrichments for (**A**) promoters, (**B**) insulators, (**C**) other predicted annotations, and (**d**) all ATAC-seq peaks. Enhancers are more enriched for relevant diseases as expected (Fig 5E) and contribute the most to disease SNP enrichments.
(TIF)

S21 Fig Runtime performance comparison for classifying *cis*-REs with CoRE-ATAC using CPU and GPU hardware. We measured the time (in seconds) to classify cis-REs using either an intel Core i9 CPU or a Tesla V100 GPU. Comparisons revealed a linear time increase as the number of peaks increases. Predictions with the CPU take ~2 minutes for 75000 peaks, which is reasonable for users of CoRE-ATAC. GPU based method comparatively take less than 20 seconds, showing the power of using a GPU for such analyses.
(TIF)
