# Supplementary figures and images for: CoRE-ATAC: A deep learning model for the functional classification of regulatory elements from single cell and bulk ATAC-seq data
Source: PLoS Comput Biol. 2021 Dec 13;17(12):e1009670. doi: 10.1371/journal.pcbi.1009670 (PMC8699717; doi:10.1371/journal.pcbi.1009670)

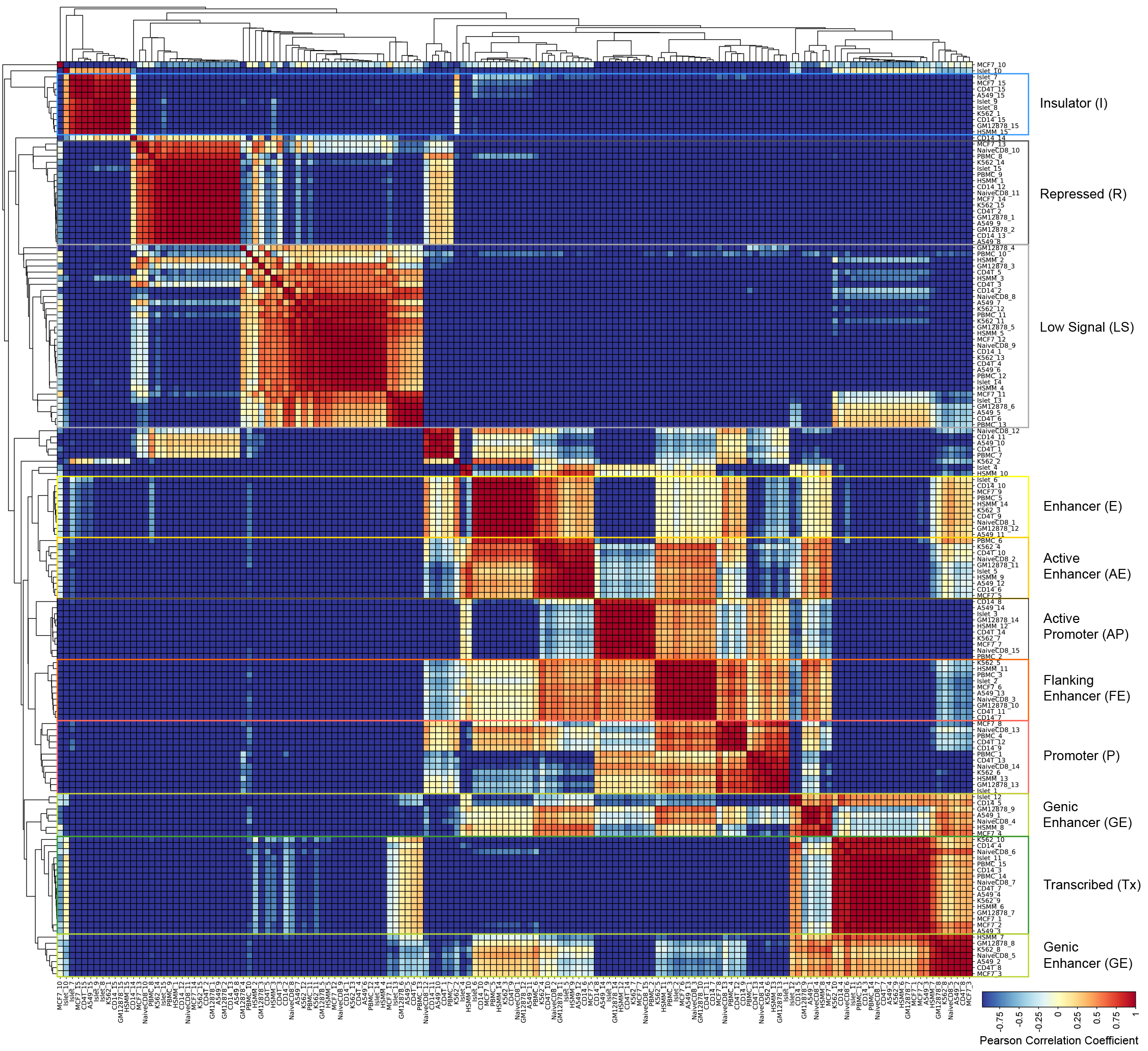

Supplement: S1 Fig — We clustered the ChromHMM emission probabilities using Pearson’s correlation coefficient to identify 10 chromatin states that were consistently present among all 10 cell types for which we recalled ChromHMM states. These 10 functional states include, insulators, repressed, low signal, enhancer, active enhancers, active promoter, flanking enhancer, promoter, genic enhancer, and transcribed regions. Numbers preceding the cell types are the emission states corresponding to S8 Fig. Note that genic enhancers are shown by two groups which correspond to active genic enhancers and genic enhancers which we combined due to the low number of cell types included within these clusters independently. Also note that for insulators, we selected islet state 7 as this was the state with the strongest CTCF signal (S8 Fig), maintaining one insulator state per cell type. (TIF) [file pcbi.1009670.s001.tif]

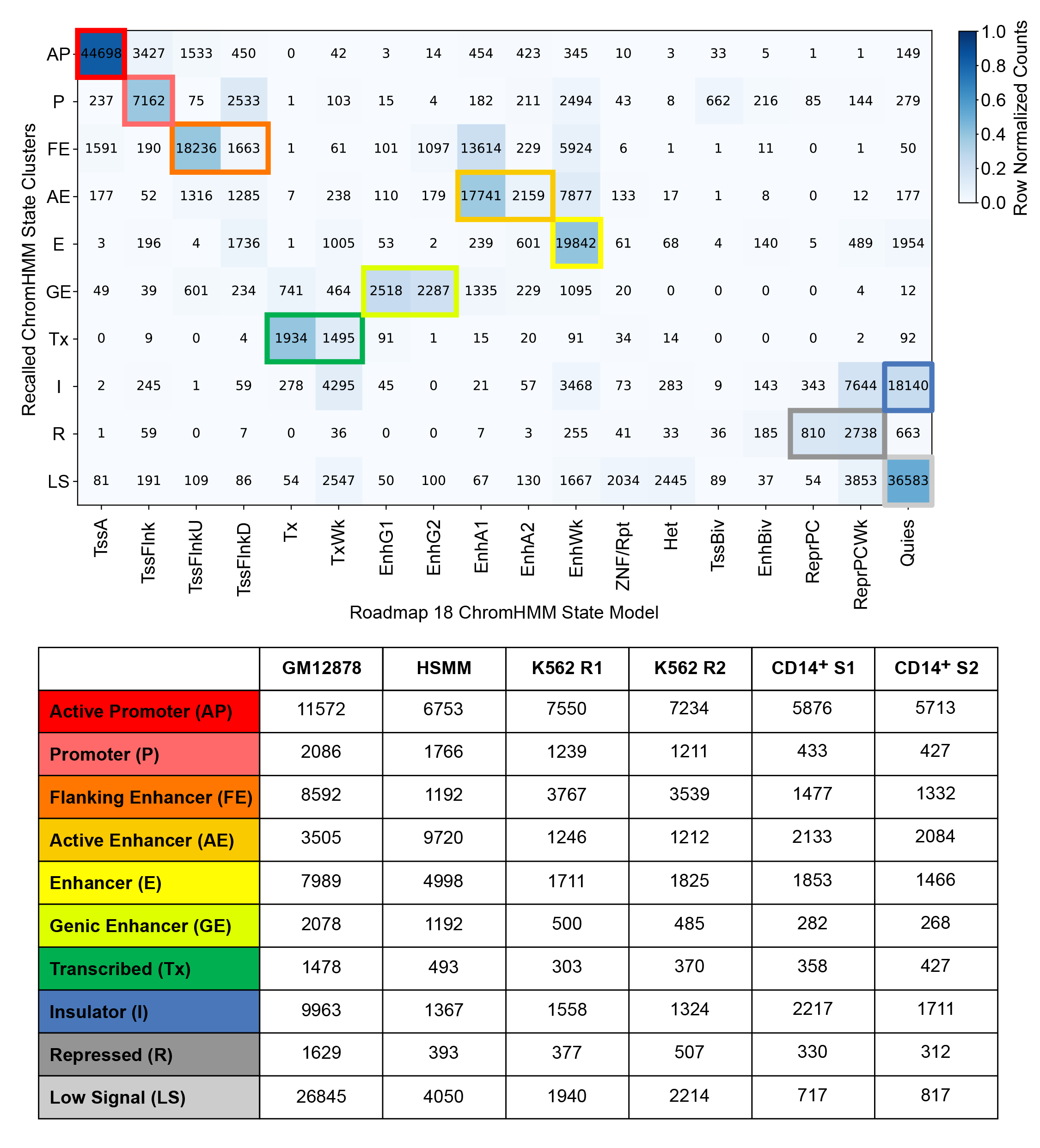

Supplement: S2 Fig — To select a ground truth for predicting the 10 functional states we identified, we corroborated our ChromHMM state calls with Roadmap 18 state models. (Top) Highlighted concordant recalled ChromHMM states with corresponding Roadmaps states to select active promoters (red), promoters (pink), flanking enhancers (orange), active enhancers (orange yellow), enhancers (yellow), genic enhancers (greenish yellow), transcribed (dark green), insulator (blue), repressed (dark gray), and low signal (light gray) functional states. (Bottom) The number of ground truth examples for each cell type used in model training and functional state examples selected. Note: Roadmap 18-state models do not include insulator states and we therefore chose insulators from our ChromHMM models that included CTCF ChIP-seq data. These predicted insulators mostly overlap with the Quiescent state in the 18-state models, suggesting that this ChromHMM state (Quies) is a miscellaneous functional state in the absence of CTCF ChIP-seq. (TIF) [file pcbi.1009670.s002.tif]

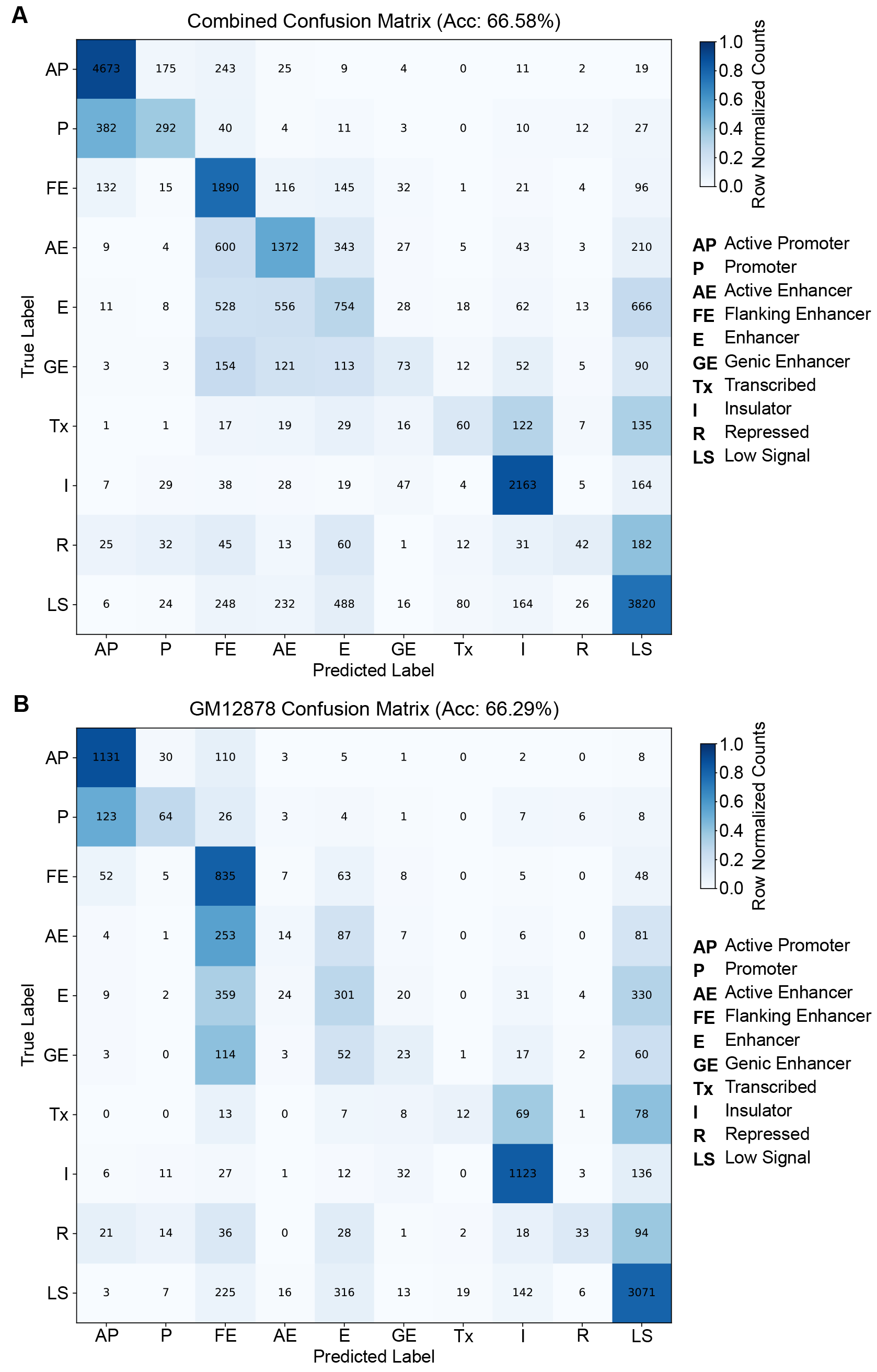

Supplement: S3 Fig — (A) Confusion matrices of combined CoRE-ATAC performances for 10 state models. (B) Confusion matrices of CoRE-ATAC performance for 10 state models in GM12878. CoRE-ATAC 10-state models. Only 4 of the 10 chromatin states were predicted by CoRE-ATAC. Smaller subsets of these four functional states (i.e., promoters, enhancers, insulators and other) were predicted as the state with the highest number of examples. (TIF) [file pcbi.1009670.s003.tif]

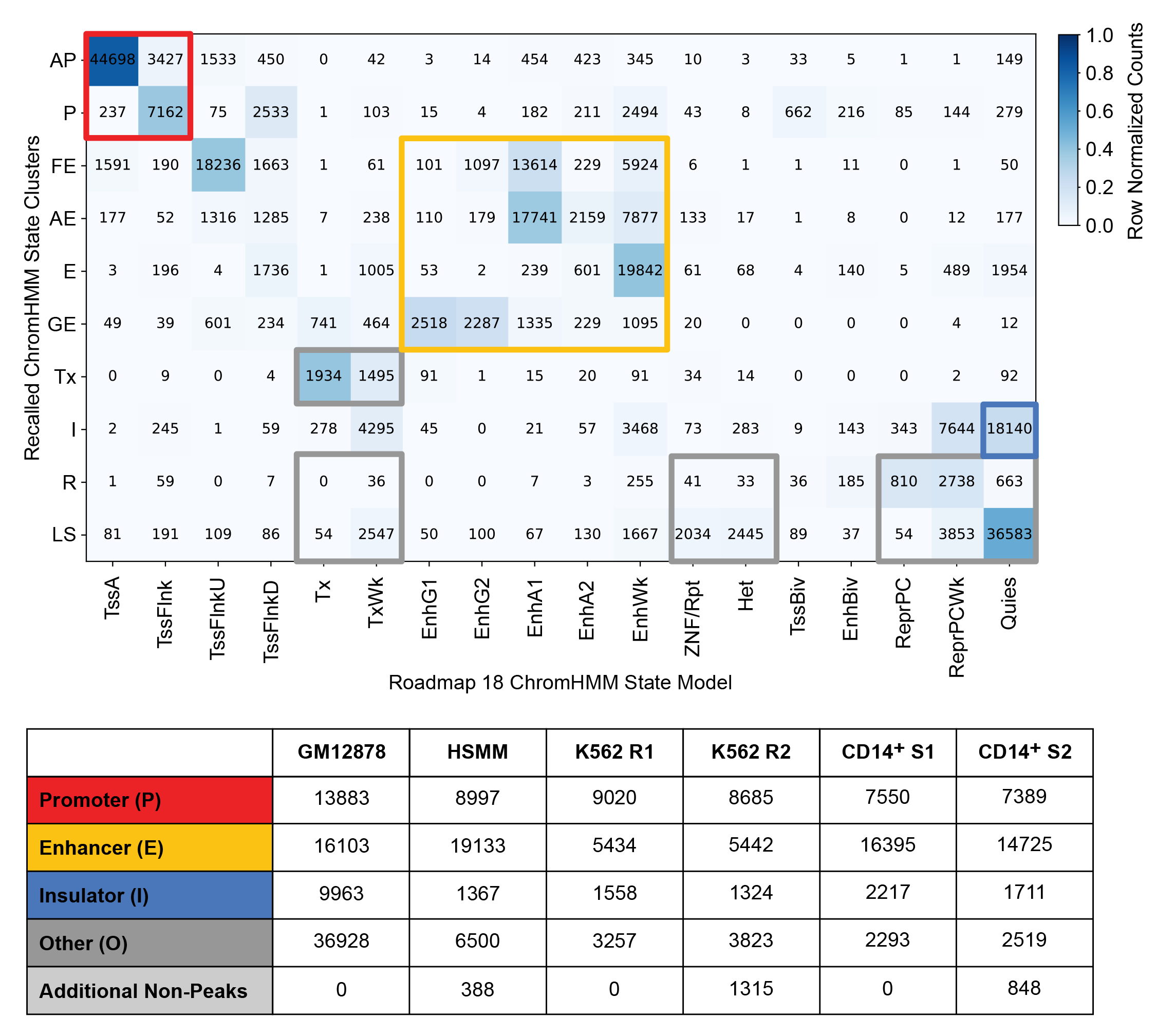

Supplement: S4 Fig — (Top) Highlighted concordant recalled ChromHMM states with corresponding Roadmaps states to select promoters, enhancers, insulators and other. (Bottom) The number of ground truth examples for each cell type used in model training and functional state examples selected. Flanking enhancers were excluded as these regions are ambiguous and could be annotated as promoters. Merging states and relaxing concordance with Roadmap allowed for selecting more examples for model training. Note: Roadmap 18-state models do not include insulator states and we therefore chose insulators from our ChromHMM models that included CTCF ChIP-seq data. These predicted insulators mostly overlap with the Quiescent state in the 18-state models, suggesting that this ChromHMM state (Quies) is a miscellaneous functional state in the absence of CTCF ChIP-seq. (TIF) [file pcbi.1009670.s004.tif]

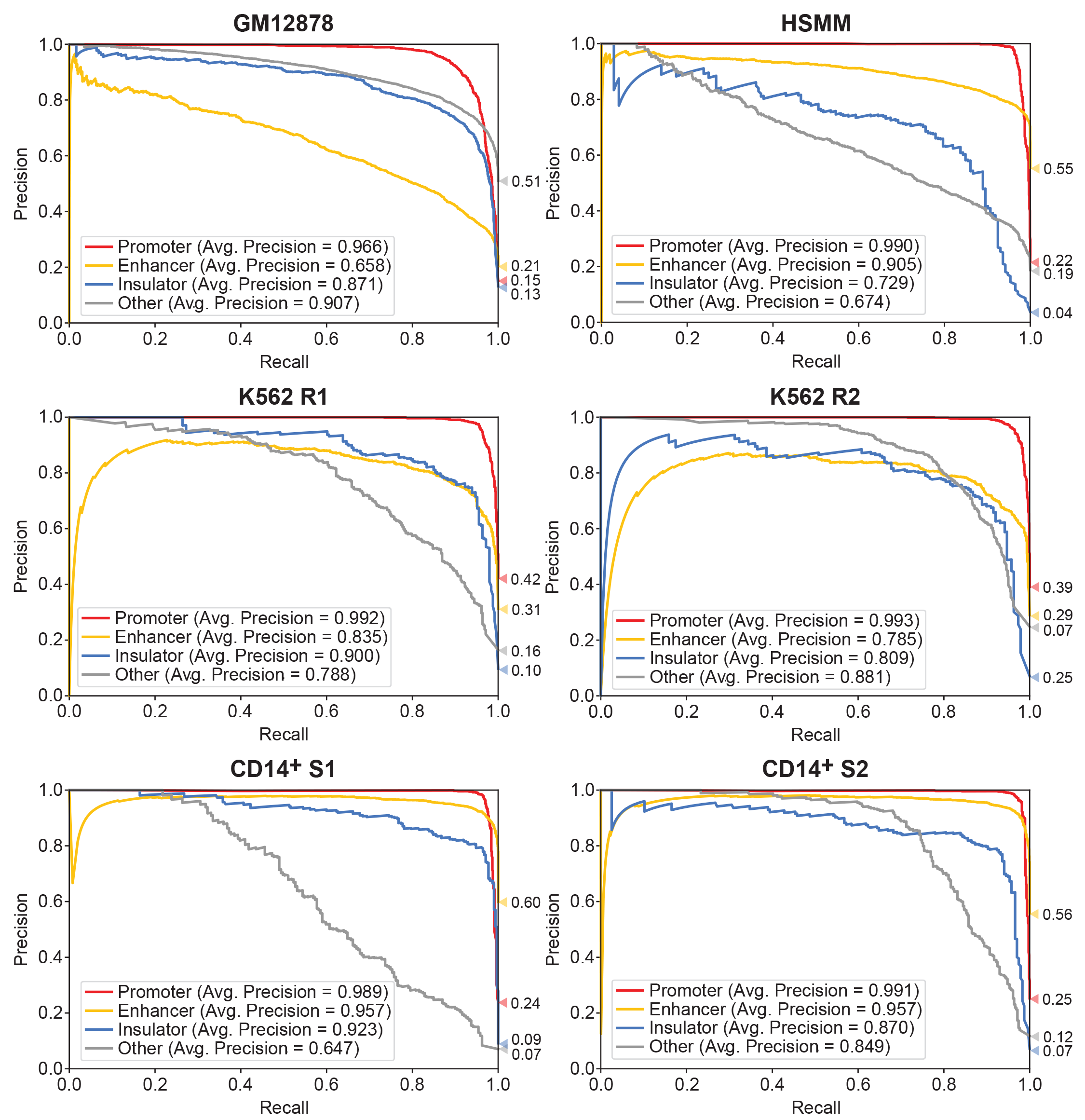

Supplement: S5 Fig — Precision recall curves of held out test data for each sample used in model training. Individual class performances reveal that CoRE-ATAC predicts all classes with high average precision. (TIF) [file pcbi.1009670.s005.tif]

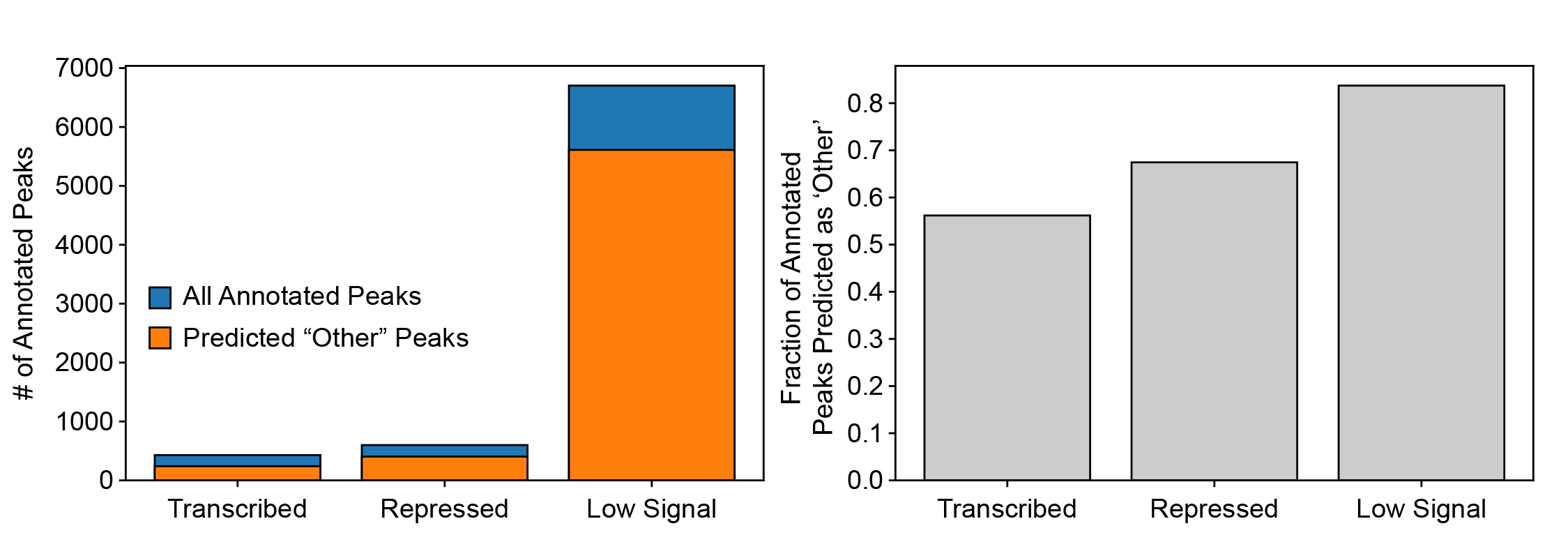

Supplement: S6 Fig — (Left) The number of peaks identified as Transcribed, Repressed, or Low Signal by ChromHMM states (blue), overlayed with the number of these peaks predicted as “Other” by CoRE-ATAC. (Right) The fraction of Transcribed, Repressed or Low Signal states annotated as “Other” by CoRE-ATAC. The majority of these states (>50% for each state, >69% overall) are predicted as “Other”. (TIF) [file pcbi.1009670.s006.tif]

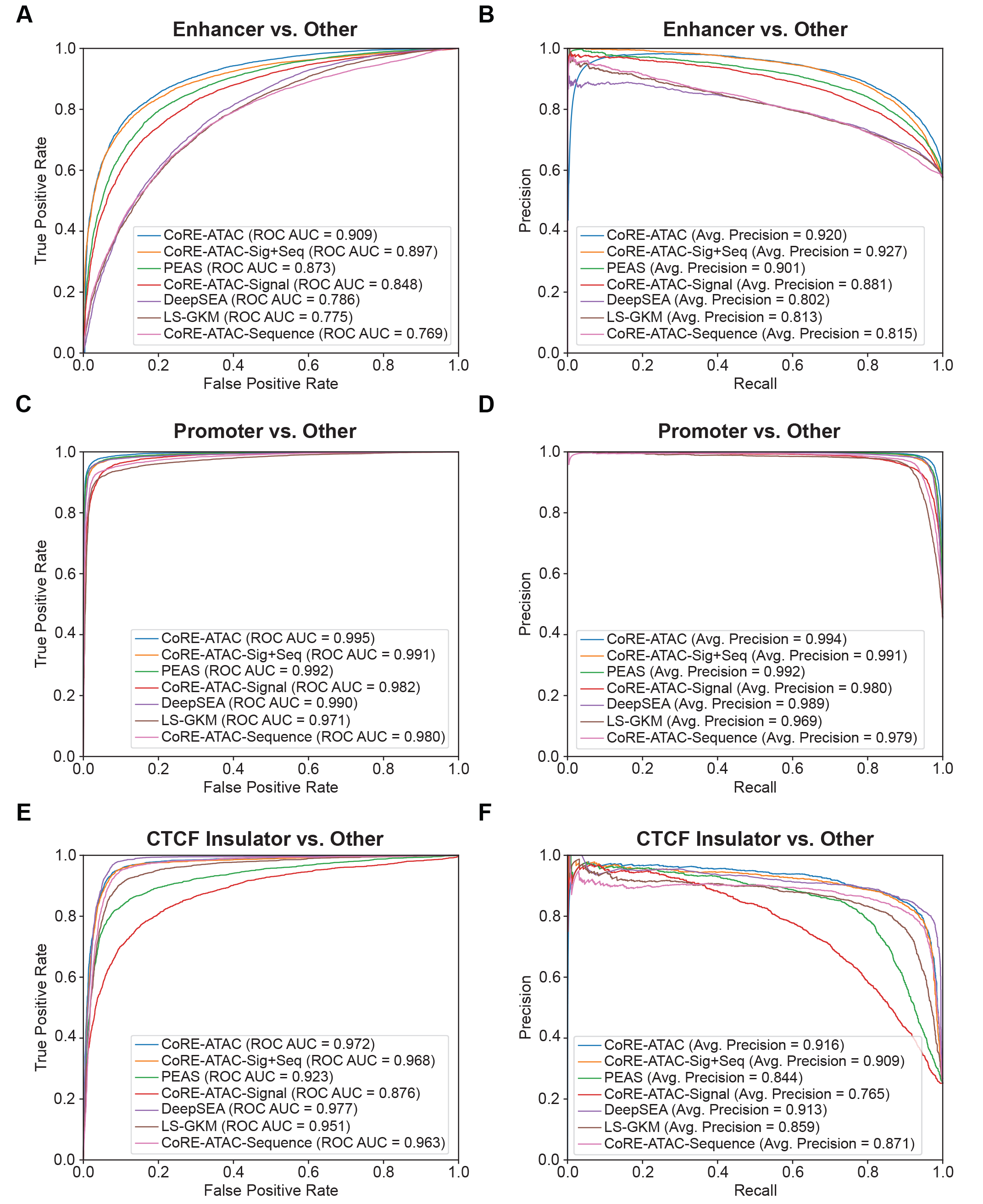

Supplement: S7 Fig — Receiver operating characteristic (ROC) curves (A) and Precision Recall curves (B) for different enhancer prediction models: CoRE-ATAC components, PEAS, DeepSEA and LS-GKM. Models were evaluated for predicting enhancer versus “other” classes for chr3 and chr11 of the GM12878, HSMM, K562, and CD14+ datasets. Sequence based approaches had similar performances, including CoRE-ATAC’s sequence-based component. The ATAC-seq signal based (CoRE-ATAC-Signal) component of CoRE-ATAC alone outperforms all sequence-based approaches, however combining both sequence and signal greatly enhances predictive performances. PEAS captures more information than signal alone, however, is still underperforming compared to CoRE-ATAC-Sig+Seq model. Finally, the CoRE-ATAC model, which includes PEAS features, showed a slight improvement over the CoRE-ATAC-Sig+Seq model, likely taking advantage of the known features such as number of known motifs and conservation scores used in PEAS. (c) ROC AUC and (d) average precision scores for predicting promoters in chr3 and chr11. All models perform with high ROC AUC and Average Precision for predicting promoters due to distinct signatures for both sequence and ATAC-eq signal. (e) ROC AUC and (f) average precision scores for predicting insulators in chr3 and chr11. DNA sequence is the approaches are the best performing models for predicting CTCF insulators, while ATAC-seq signal alone provides the weakest predictive power for annotating these cis-REs. (TIF) [file pcbi.1009670.s007.tif]

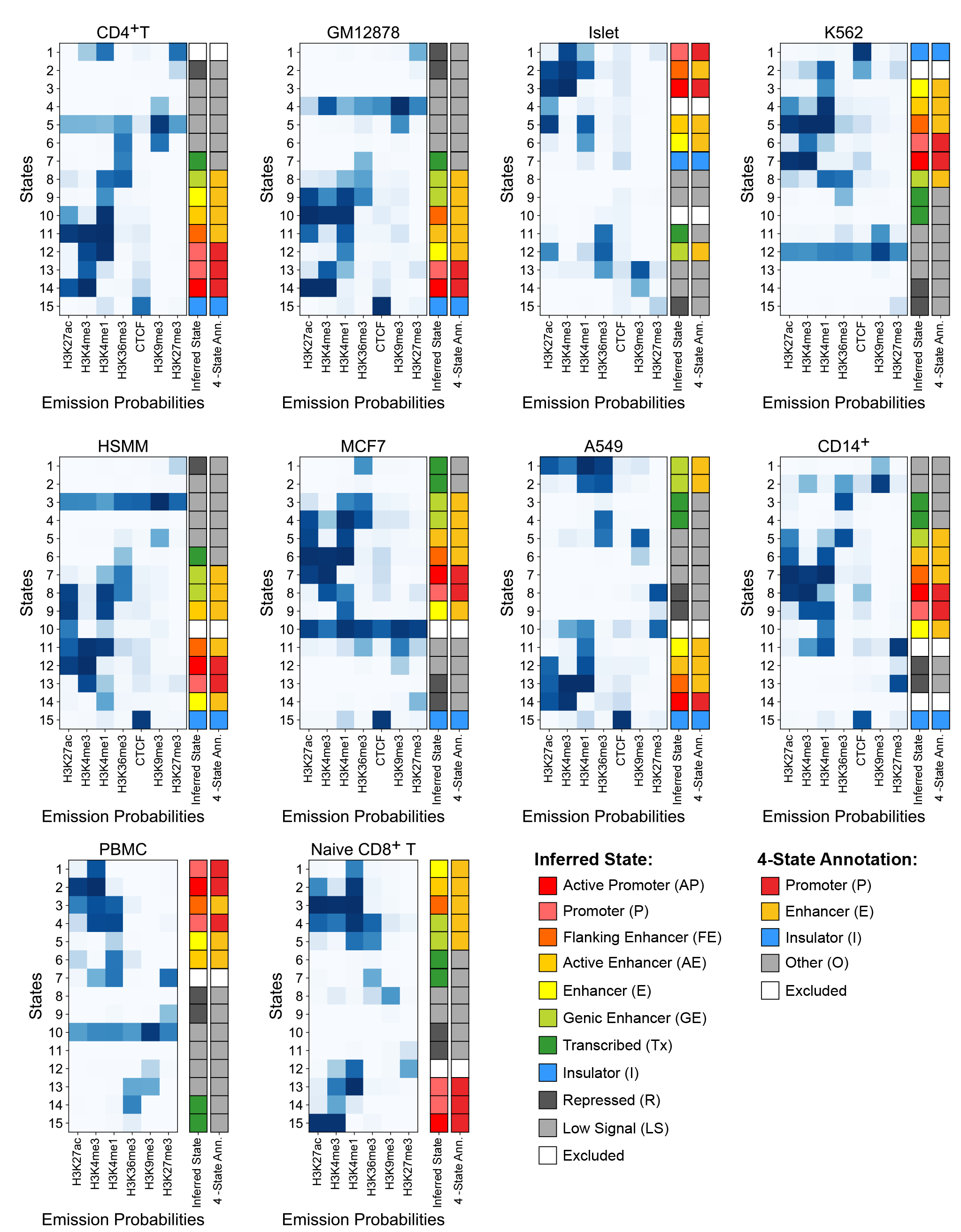

Supplement: S8 Fig — Recalled ChromHMM states for 10 of the 11 cell types used in this study. Each chromHMM run included enhancer marker H3k4me1, promoter marker H3k4me3, repressor marker H3k27me3, active cis-RE marker H3k27ac, transcribed marker H3k36me3, heterochromatin marker H3k9me3, and CTCF insulator marker CTCF (when available). Emission probabilities revealed consistent histone modification mark combinations present throughout these diverse cell types. Color annotations next to each heatmap of ChromHMM states represent the inferred ChromHMM state and the final 4 state relabeling. (TIF) [file pcbi.1009670.s008.tif]

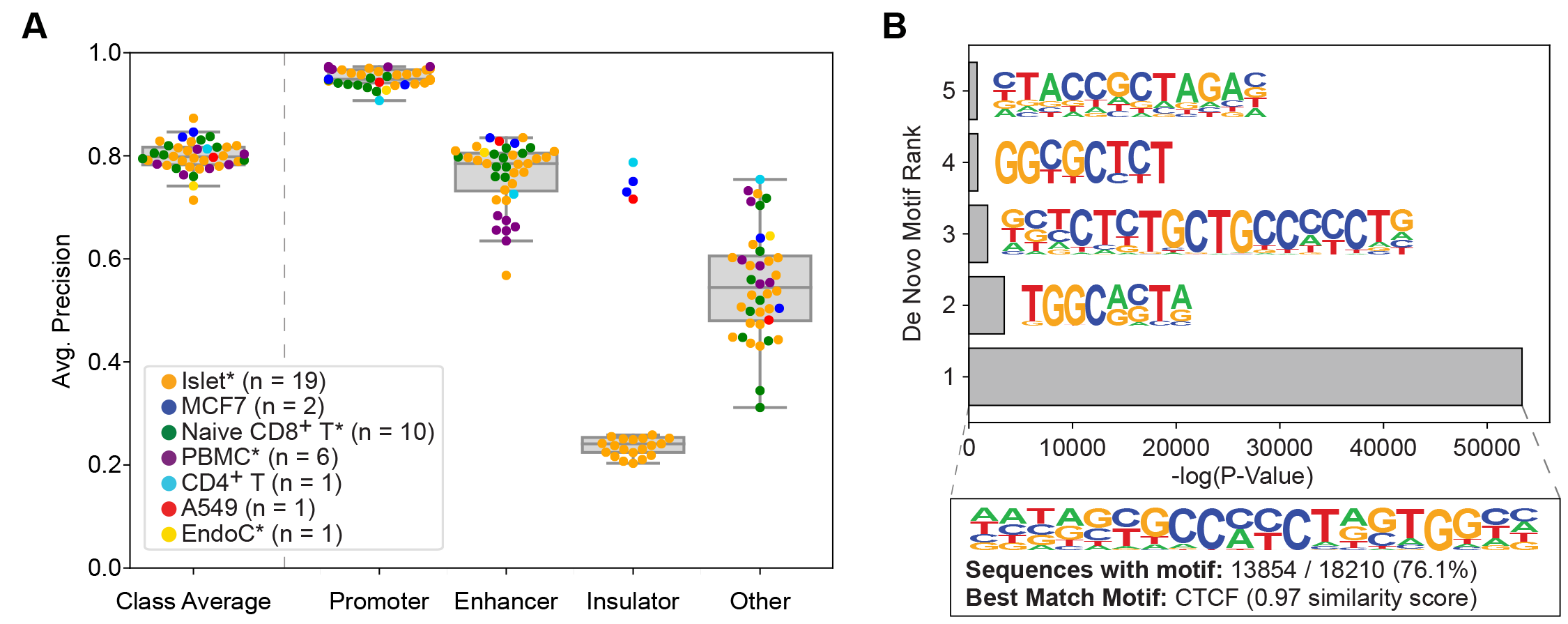

Supplement: S9 Fig — (A) Cross cell type model performances when including islet insulators. Using poor quality CTCF ChIP-seq data (as evident from S8 Fig) as a ground truth resulted in reduced model performances for all islet samples. (B) De novo motif enrichment for CoRE-ATAC insulator predictions in islets. Predicted insulators are highly enriched for CTCF with 76.1% of regions harboring a CTCF motif. Incorporating both DNA sequence and ATAC-seq signal enables CoRE-ATAC to detect a majority of insulators using the CTCF motif while detecting other insulators via other features learned in model training. (TIF) [file pcbi.1009670.s009.tif]

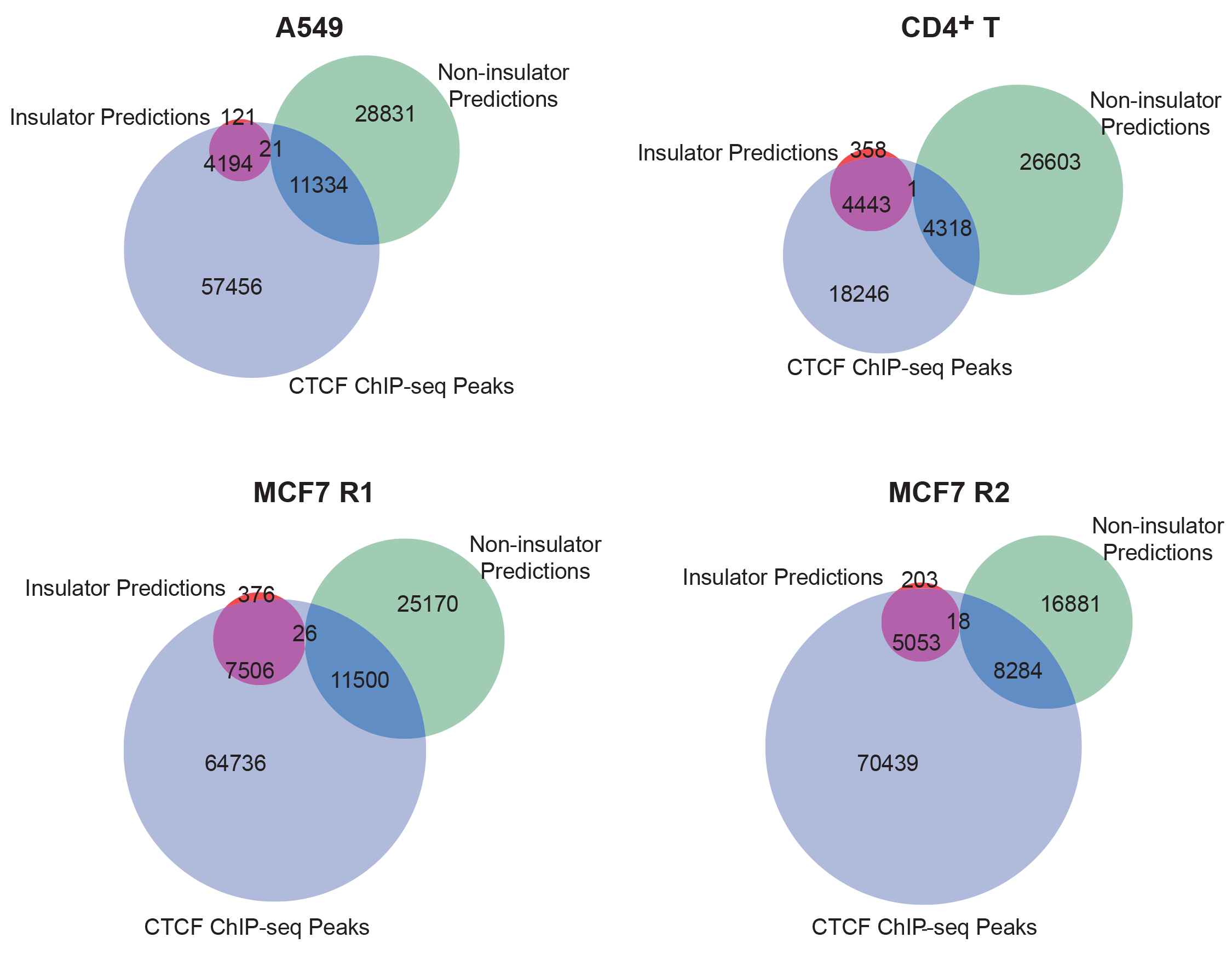

Supplement: S10 Fig — Overlap of CTCF ChIP-seq peaks with CoRE-ATAC insulator and non-insulator predictions. Majority of CoRE-ATAC insulator predictions overlapped with CTCF ChIP-seq peaks. Note: Overlaps between insulator and non-insulators are the result of CTCF peaks bridging the gap between these genomic regions when merging peaks for the union. (TIF) [file pcbi.1009670.s010.tif]

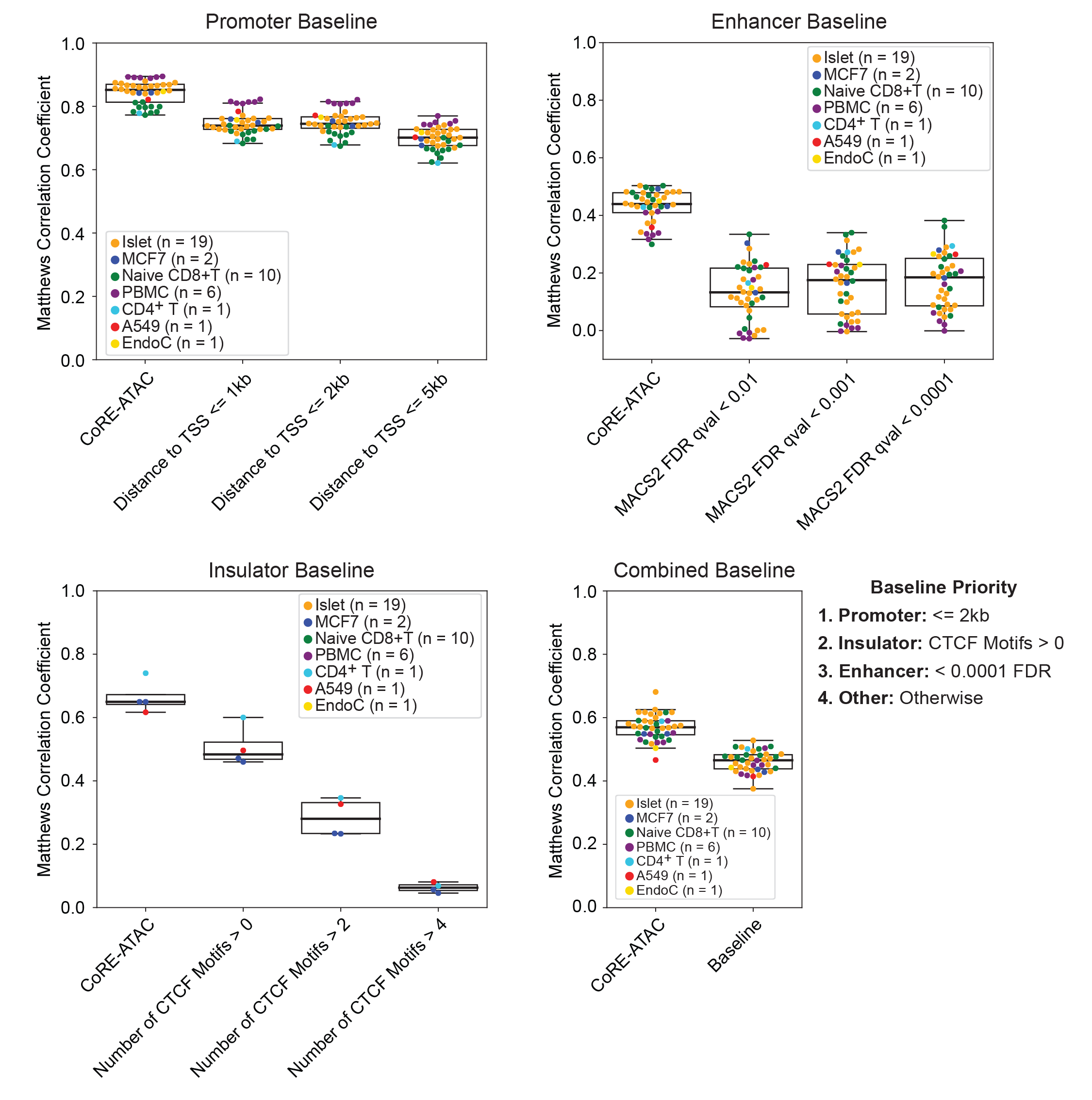

Supplement: S11 Fig — CoRE-ATAC outperforms each threshold-based method for detecting promoters, enhancers, insulators, and combined annotations. Matthews Correlation Coefficient was used to measure performances as it accounts for true positives, true negatives, false positives, and false negatives simultaneously within its function and is an ideal measurement for performances when probabilities are static as in the case with all threshold-based approaches (i.e., probability = 1.0 if satisfying the threshold, 0.0 otherwise). CoRE-ATAC consistently had the best performance compared to each baseline. For detecting promoters, the best threshold was identified as ATAC-seq peaks within 2kb of the promoter, for enhancers, an FDR qval score is less than 0.0001, and for insulators, whether or not the region contained a single (n = 1) CTCF motif was the best performing threshold. Finally, combining all three of these thresholds confirmed that CoRE-ATAC improves our ability to predict cis-RE function, outperforming commonly used methods for detecting promoters, enhancers, and insulators. (TIF) [file pcbi.1009670.s011.tif]

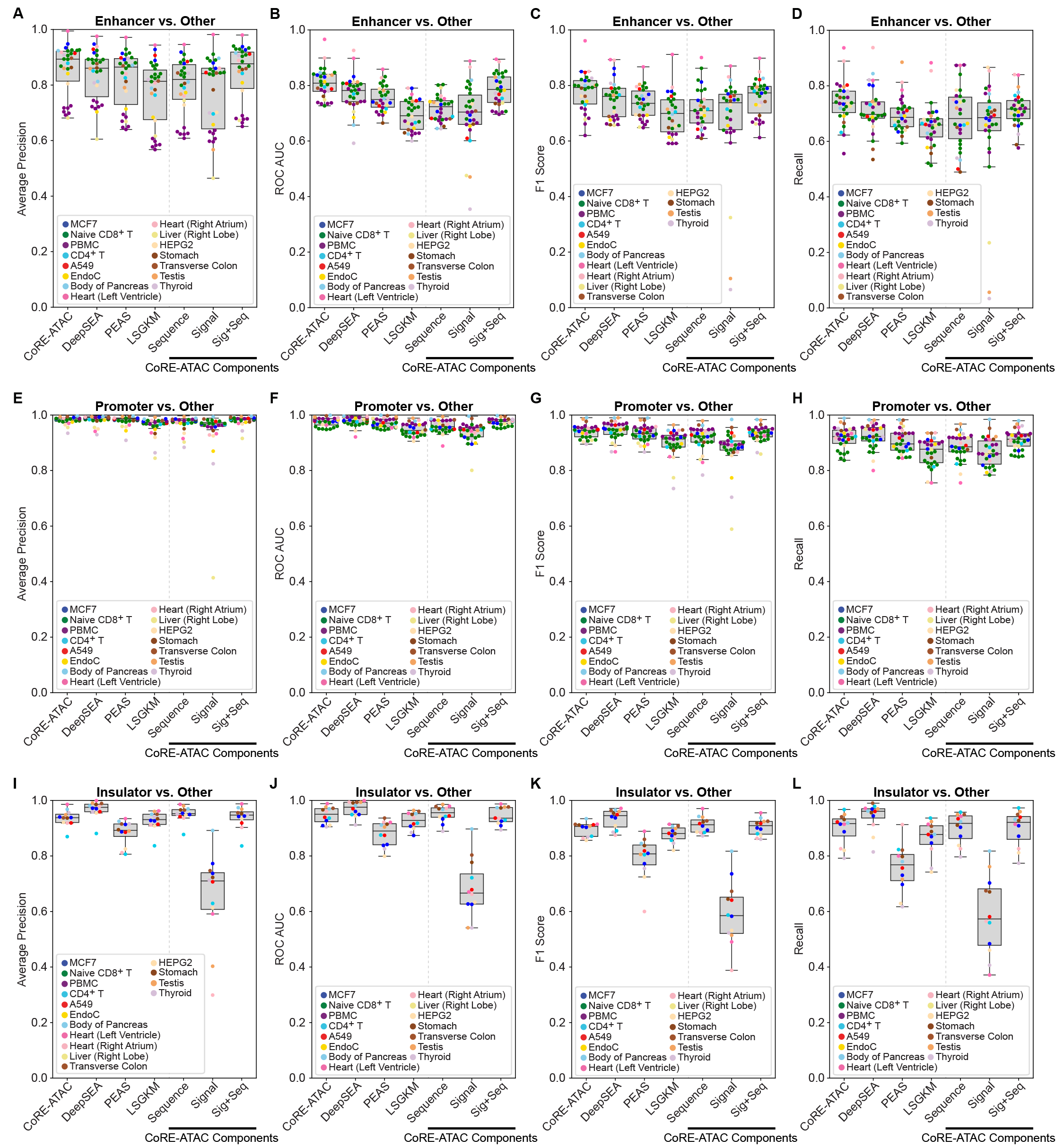

Supplement: S12 Fig — (A) ROC AUC, (B) average precision, (C) F1, and (D) recall scores for predicting enhancers in chr3 and chr11 across different cell types not used in model training. CoRE-ATAC generalizes well for predicting enhancers in new cell types. CoRE-ATAC outperforms alternative sequence-based models (Mann Whitney P-Values over ROC AUC < 0.0096). Note: The model for DeepSEA was trained using MCF7, A549, Stomach, Heart Left Ventricle, Heart Right Atrium, Pancreas, Liver, HEPG2, CD8 Naïve T Cells, and CD4+ T cells.(E-H) Performance scores for predicting promoters in chr3 and chr11 across different cell types not used in model training. All models predict promoters with high accuracy, irrespective of cell type. (I-L) Performance scores for predicting insulators in chr3 and chr11 across different cell types not used in model training. DeepSEA outperforms other methods for detecting CTCF insulators. CTCF has a well characterized DNA binding sequence that is very predictive. (TIF) [file pcbi.1009670.s012.tif]

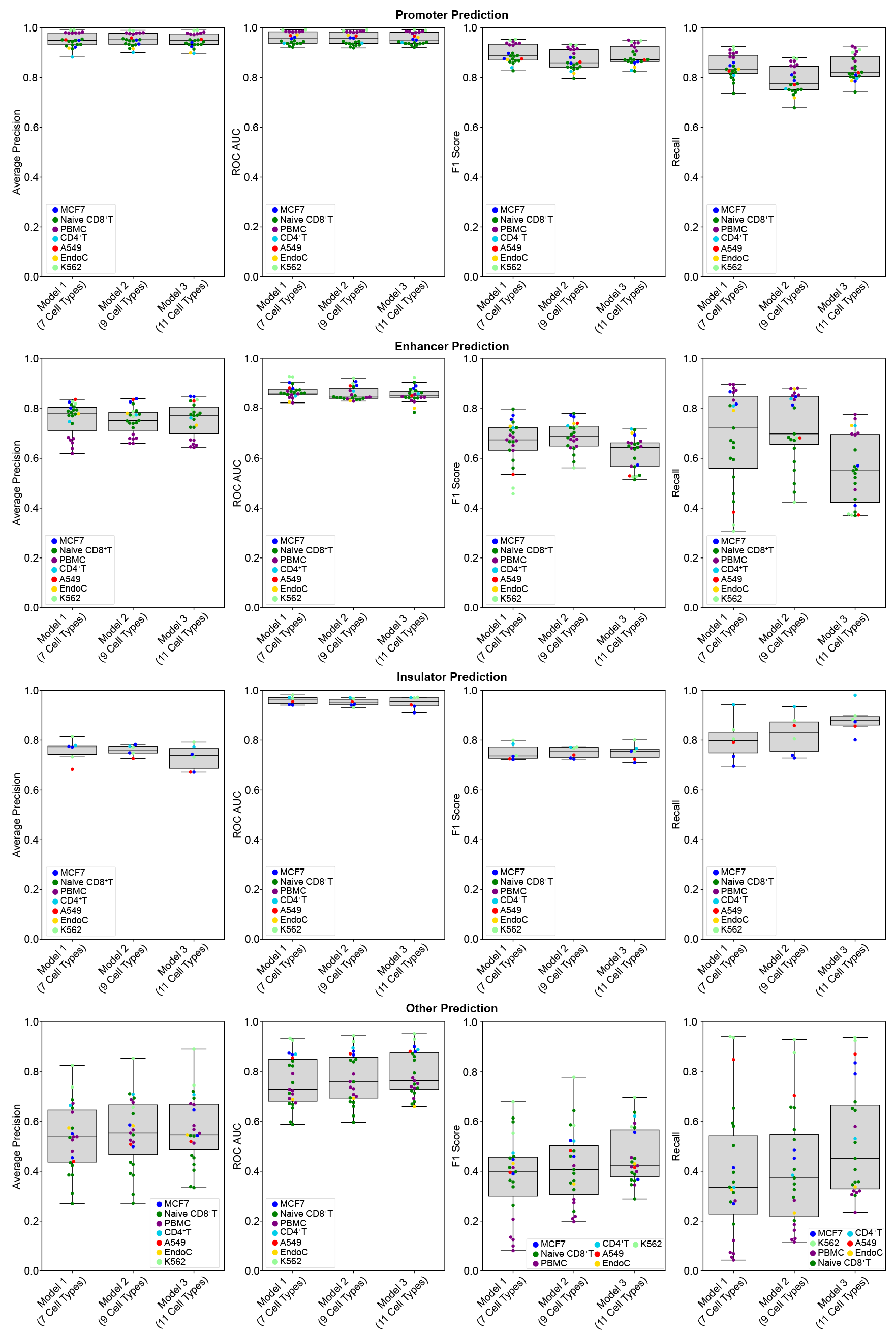

Supplement: S13 Fig — Three CoRE-ATAC models were trained using: 1) 7 different cell types: GM12878, HSMM, Pancreas, Stomach, Thyroid, Testis, and Transverse Colon, 2) 9 different cell types: 7 cell type model with 2 Heart samples (Left Ventricle and Right Atrium Auricular Region) and 3) 11 different cell types: 9 cell type model with Liver and HepG2 samples. CoRE-ATAC performances did not significantly improve with increased number of cell types used for training for promoter, enhancer, or other prediction. Insulator prediction showed significant differences between models (Mann Whitney P-Values < 1.91e-06, Average Precision), however, the effect of these differences suggests that the model decreased in performance as more data was used for model training. (TIF) [file pcbi.1009670.s013.tif]

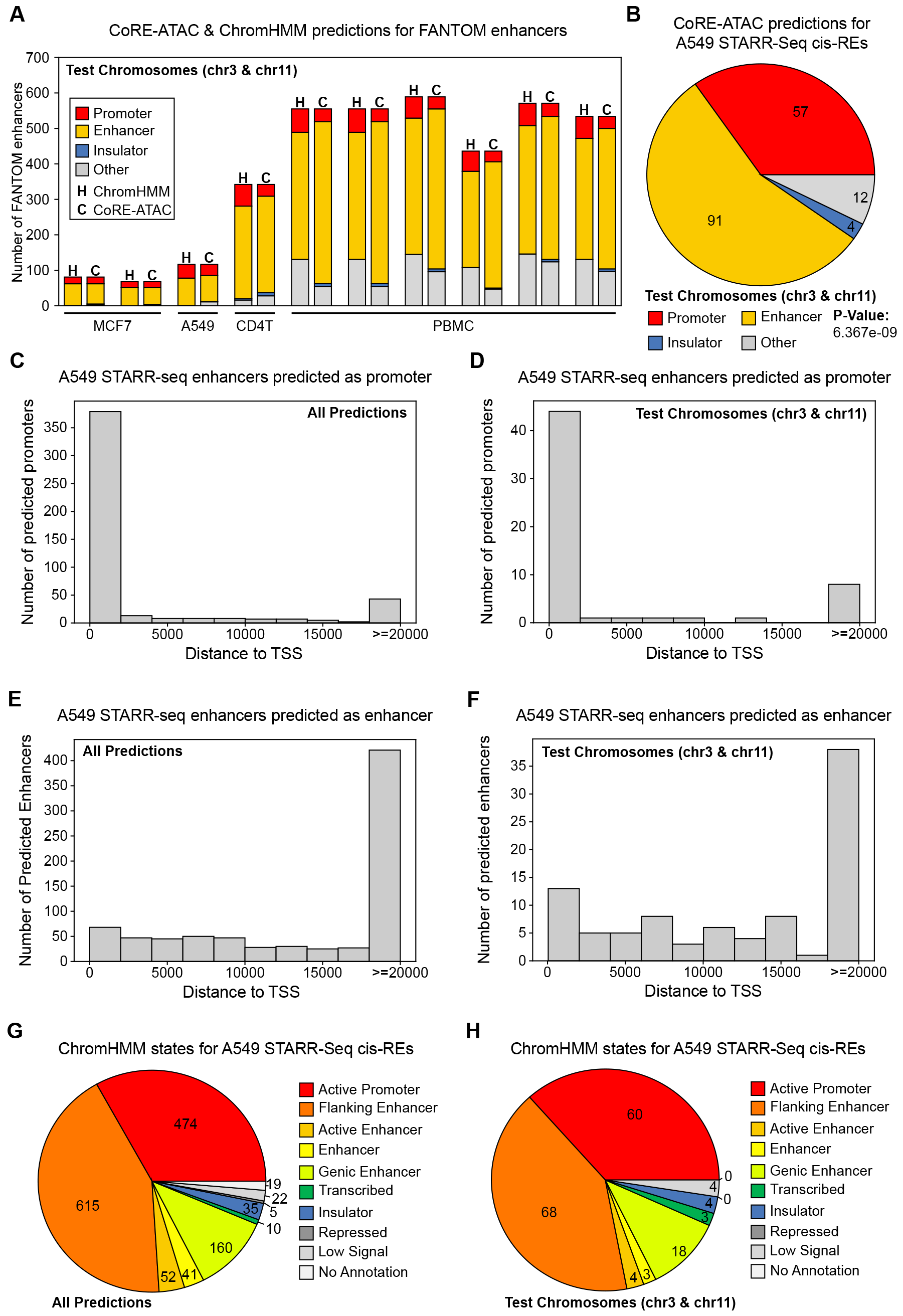

Supplement: S14 Fig — (A) Distribution of CoRE-ATAC predictions for FANTOM enhancers in test chromosomes (chr3 and chr11). (B) Distribution of CoRE-ATAC predictions for test chromosomes. Pairs of bars represent comparisons between ChromHMM (H) and CoRE-ATAC (C), where each pair represents a sample/replicate for the respective cell type. (C,D) Histogram of distances to the nearest TSS for STARR-seq enhancers predicted as promoters by CoRE-ATAC for all chromosomes (C) and test chromosomes (D). Majority of predicted promoters are within 1kb of a TSS. (e,f) Histogram of distances to the nearest TSS for STARR-seq enhancers predicted as enhancers by CoRE-ATAC for all chromosomes (E) and test chromosomes (F). Majority of predicted enhancers are distal (> = 20kb) from the nearest TSS. STARR-seq enhancers annotated as promoters result from the close proximity these enhancers are to a TSS. (G,H) ChromHMM annotation (relabeled to 10 classes) distribution for STARR-seq enhancers in A549 for all chromosomes (G) and test chromosomes (H). Majority of STARR-seq enhancers are annotated as promoter or enhancer by ChromHMM. (TIF) [file pcbi.1009670.s014.tif]

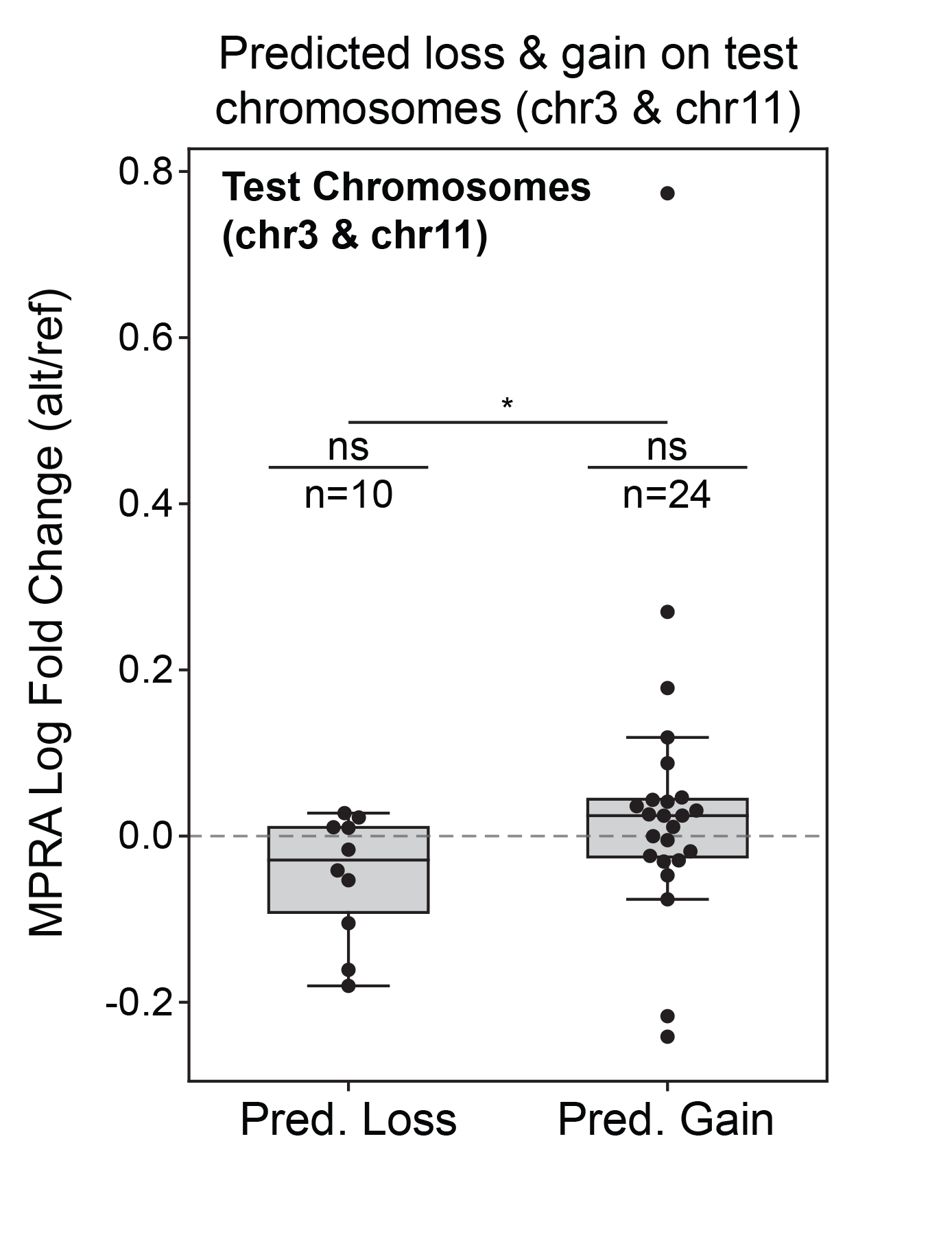

Supplement: S15 Fig — MIN6 MPRA log fold change values for genomic regions predicted as losing or gaining cis-RE function based on CoRE-ATAC probabilities for reference and alternative alleles in test chromosomes (chr3 and chr11). Significance for predicted loss and predicted gain categories was calculated using student’s t-test for MPRA log fold change values being less than or greater than 0 respectively. Significance comparing the predicted loss and predicted gain of MPRA fold change distributions was calculated using Mann-Whitney U test. Concordant direction of effect (P-Value < 0.022) was observed for both for CoRE-ATAC predictions and MPRA activity levels for chromosomes not used in model training. (TIF) [file pcbi.1009670.s015.tif]

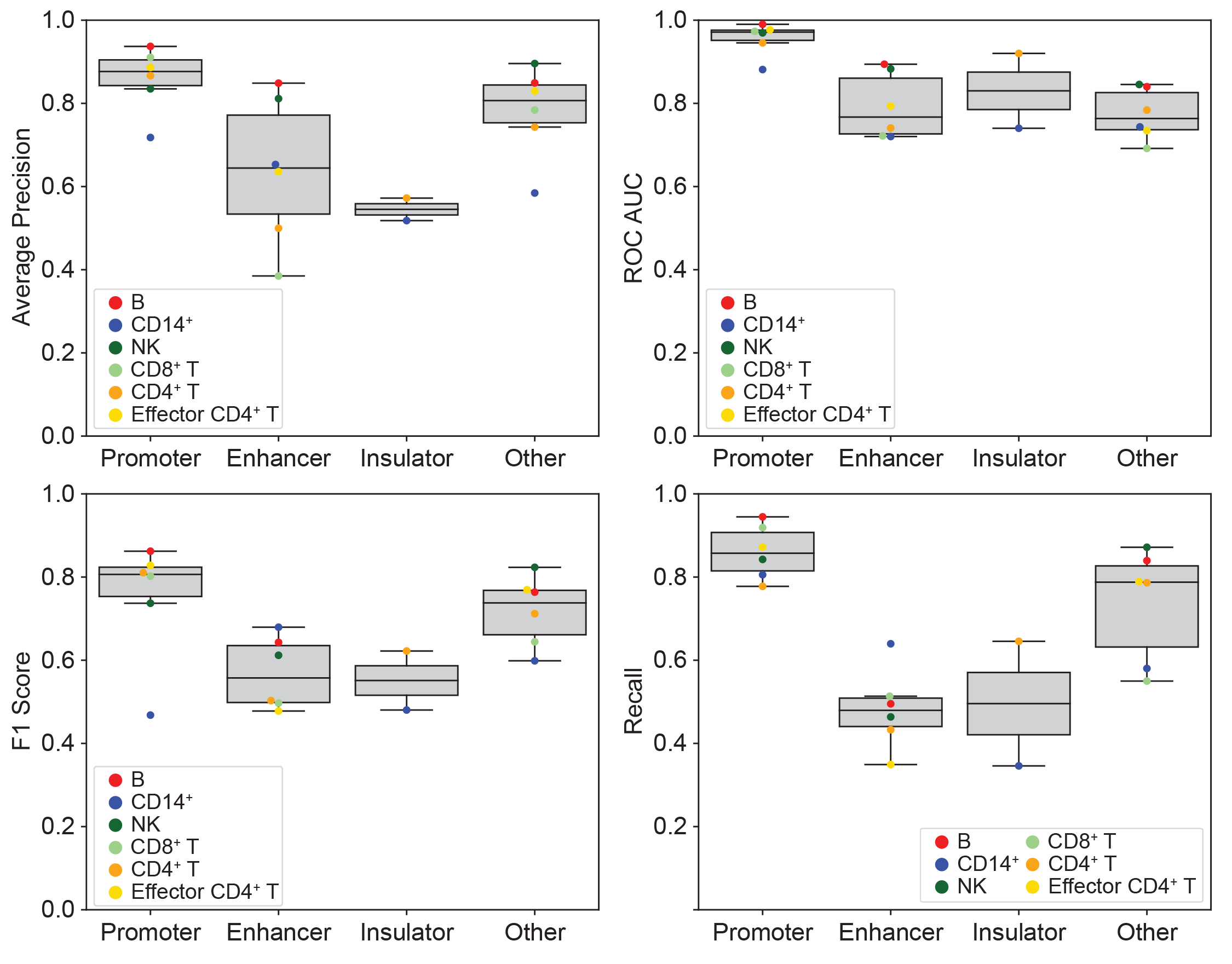

Supplement: S16 Fig — Average precision, ROC AUC, F1 score, and recall for predicting cis-RE function in snATAC for 6 annotated clusters with available ChromHMM states. Model performances suggest that CoRE-ATAC is an effective tool for interrogating cis-RE activity from snATAC data. (TIF) [file pcbi.1009670.s016.tif]

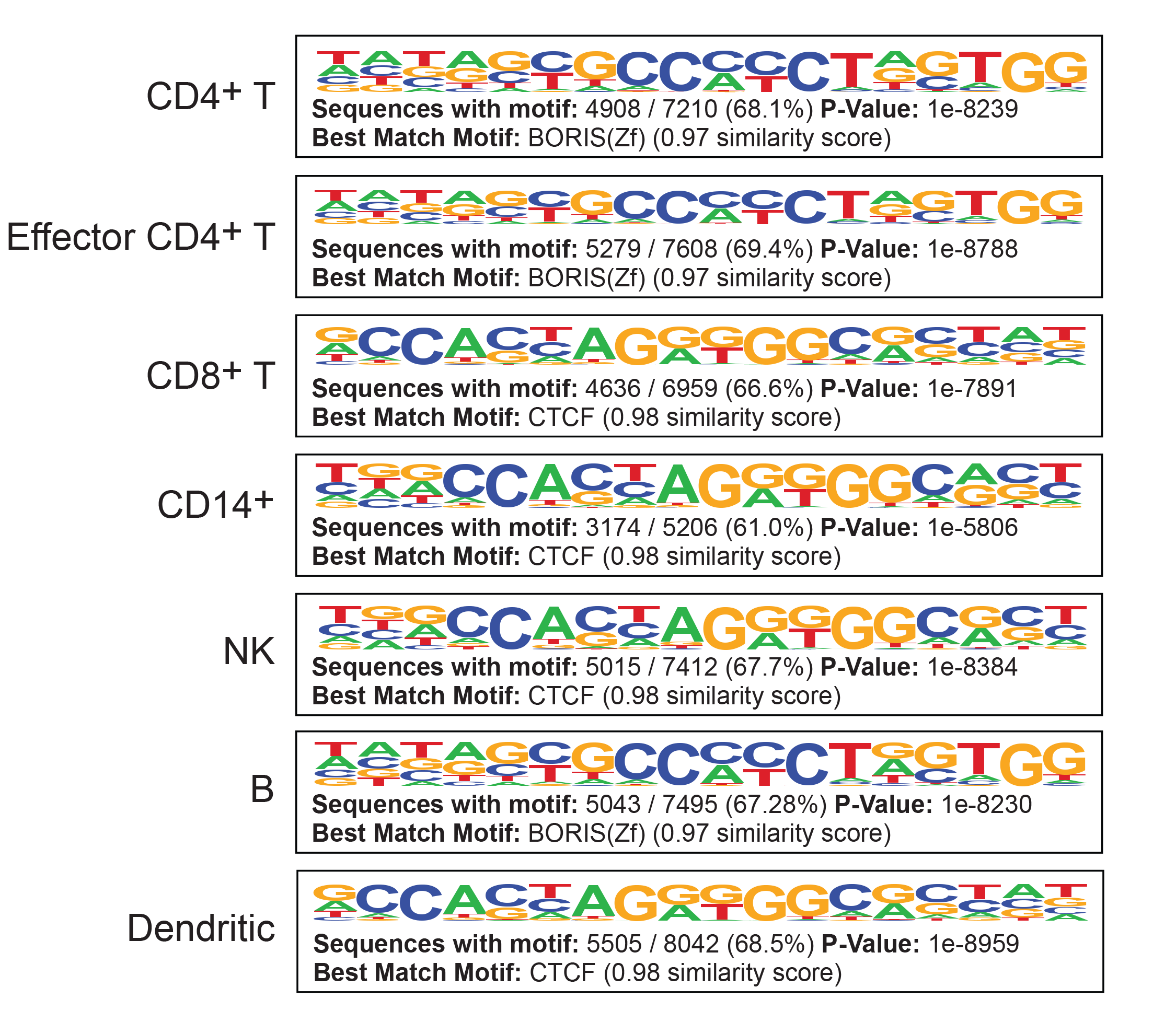

Supplement: S17 Fig — Top de novo motif enrichments for all seven cell type clusters for snATAC data. Insulator predictions for all cell types are significantly enriched for motifs with high similarity with CTCF. (TIF) [file pcbi.1009670.s017.tif]

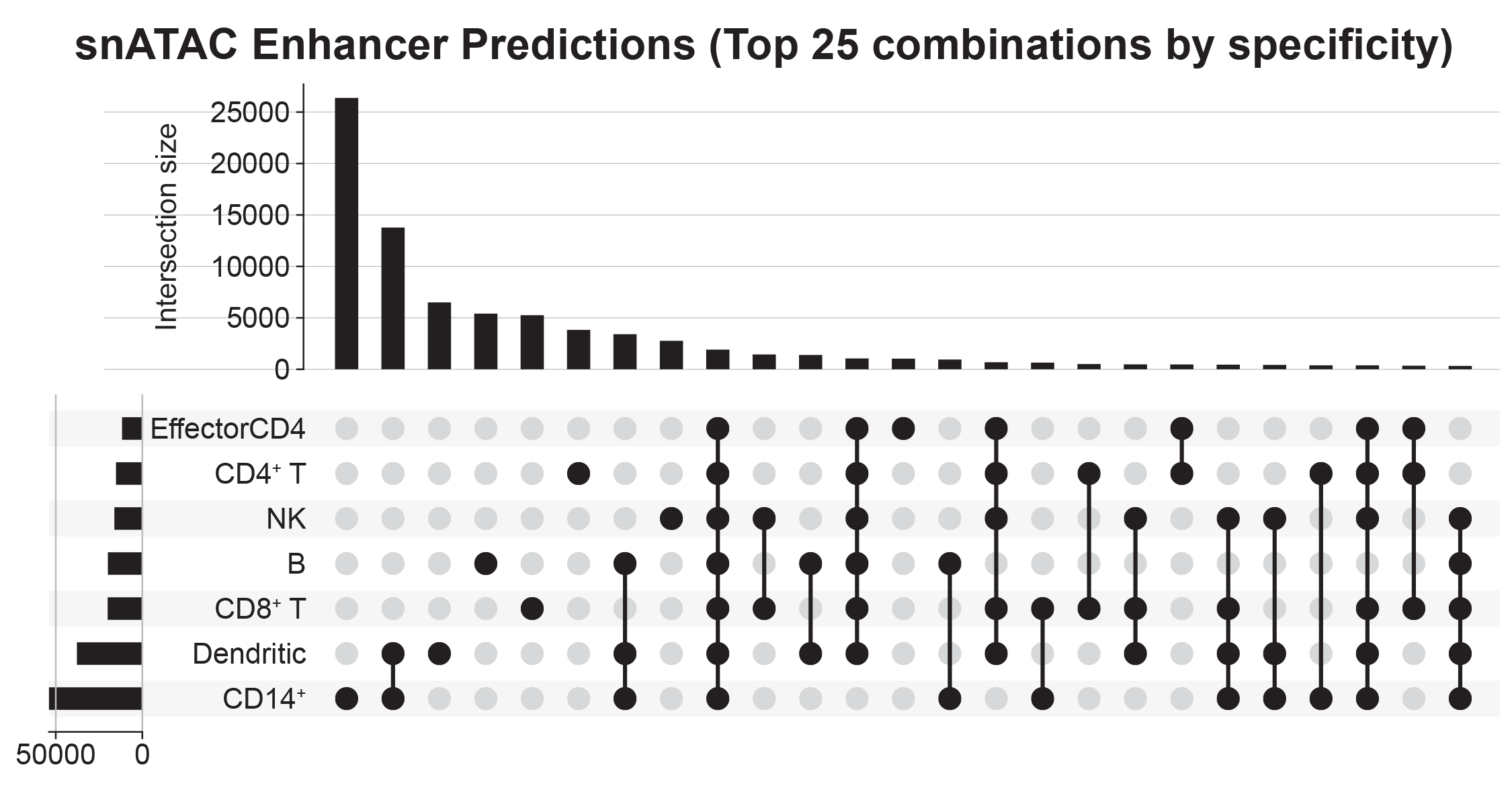

Supplement: S18 Fig — Number of enhancers identified for the top 25 cell types and combinations by the number of enhancers predicted by CoRE-ATAC. (TIF) [file pcbi.1009670.s018.tif]

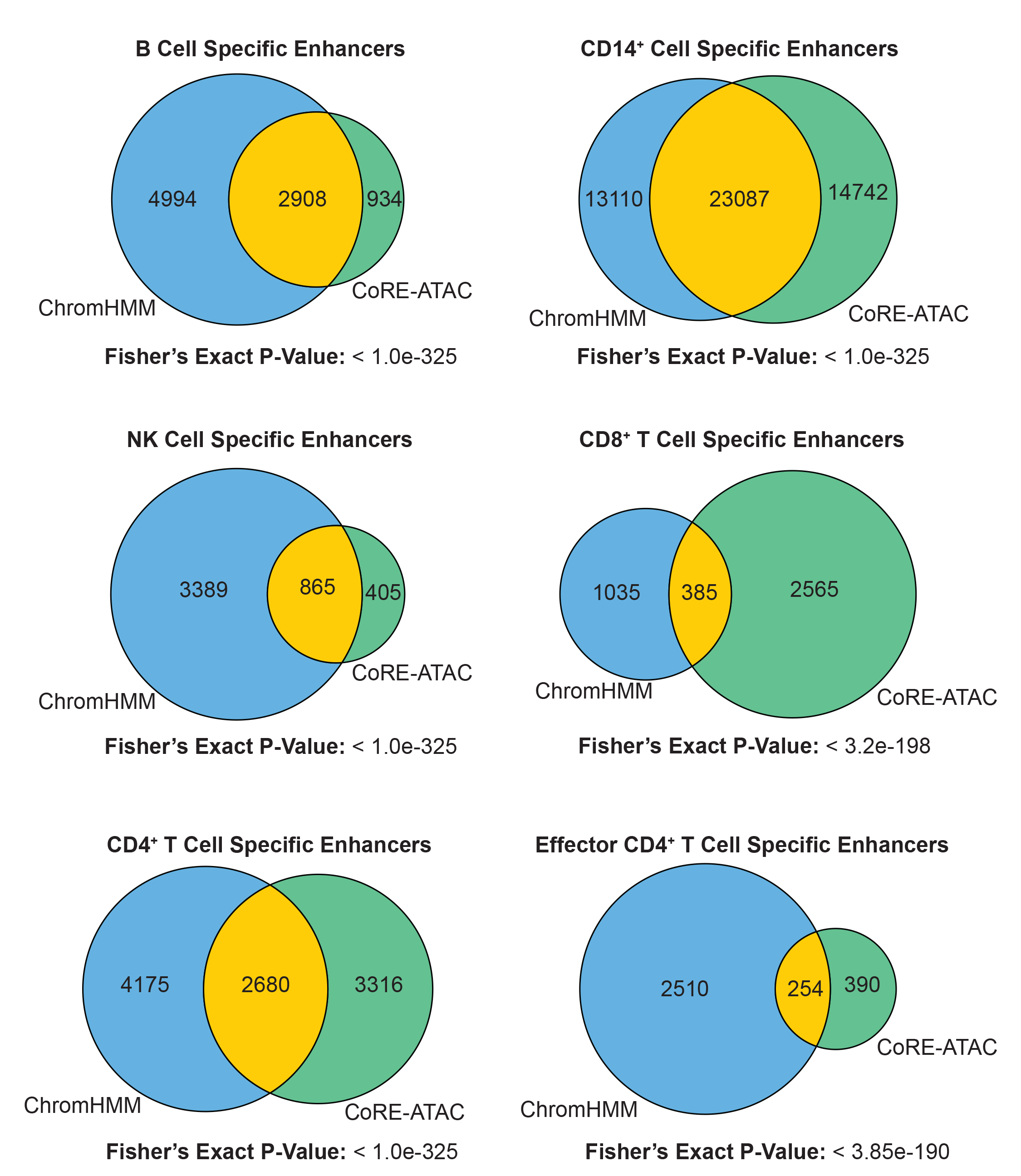

Supplement: S19 Fig — Overlap of CoRE-ATAC andChromHMM cell-specific enhancers. CoRE-ATAC predictions significantly overlap ChromHMM enhancers for all cell types (P-Values < 3.85e-190 Fisher exact test). Overlaps were less significant for T cell comparisons, partially due to the differences in sorted cells used in ChromHMM annotations and total cells used in CoRE-ATAC predictions from snATAC-seq data. (TIF) [file pcbi.1009670.s019.tif]

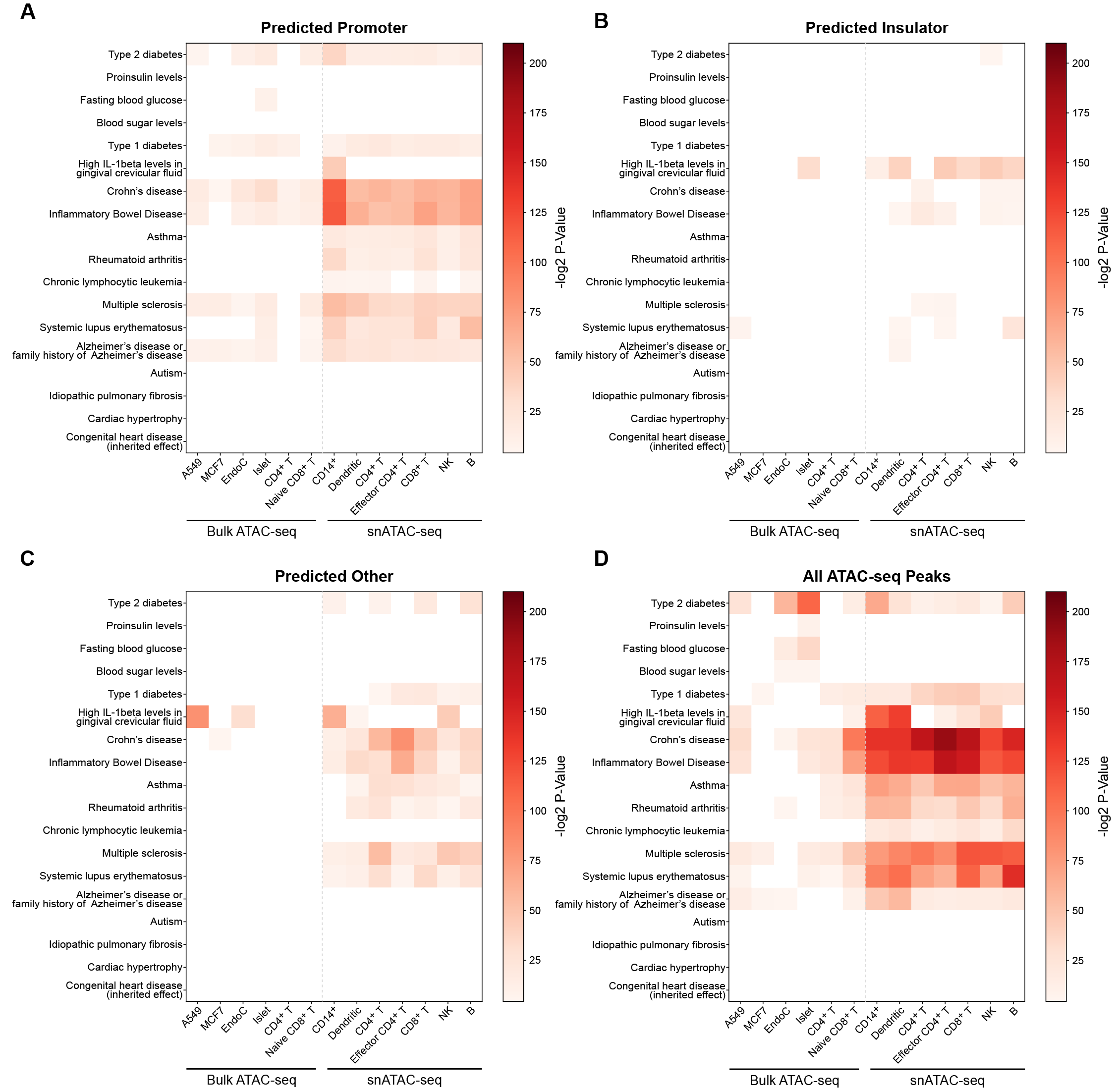

Supplement: S20 Fig — Selected disease SNP enrichments for (A) promoters, (B) insulators, (C) other predicted annotations, and (d) all ATAC-seq peaks. Enhancers are more enriched for relevant diseases as expected (Fig 5E) and contribute the most to disease SNP enrichments. (TIF) [file pcbi.1009670.s020.tif]

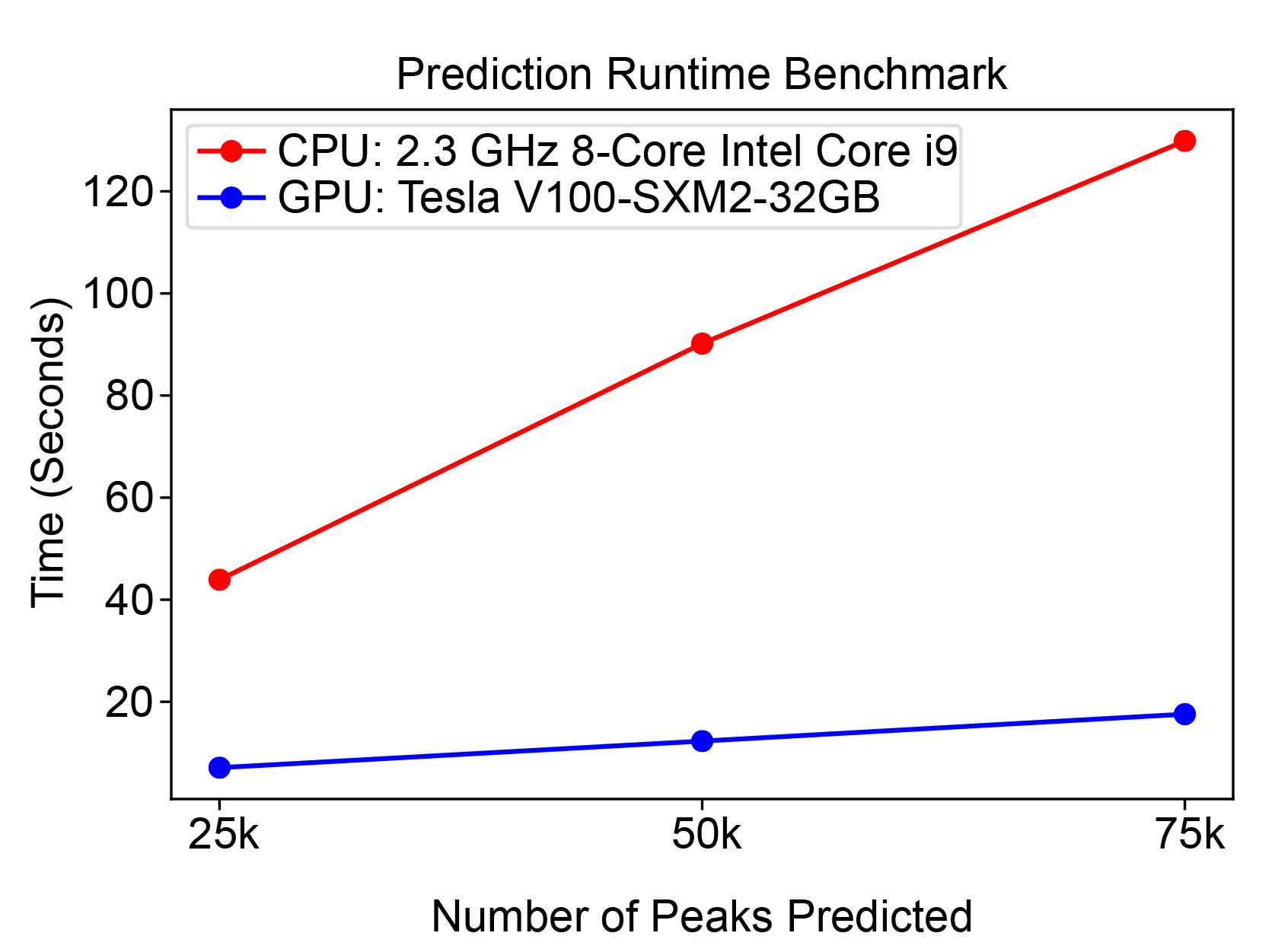

Supplement: S21 Fig — We measured the time (in seconds) to classify cis-REs using either an intel Core i9 CPU or a Tesla V100 GPU. Comparisons revealed a linear time increase as the number of peaks increases. Predictions with the CPU take ~2 minutes for 75000 peaks, which is reasonable for users of CoRE-ATAC. GPU based method comparatively take less than 20 seconds, showing the power of using a GPU for such analyses. (TIF) [file pcbi.1009670.s021.tif]
